# Genome Analysis and Replication Studies of the African Green Monkey Simian Foamy Virus Serotype 3 Strain FV2014

**DOI:** 10.3390/v12040403

**Published:** 2020-04-06

**Authors:** Sandra M. Fuentes, Eunhae H. Bae, Subhiksha Nandakumar, Dhanya K. Williams, Arifa S. Khan

**Affiliations:** 1 Laboratory of Retroviruses, Division of Viral Products, Office of Vaccines Research and Review, Center for Biologics Evaluation and Review, U.S. Food and Drug Administration, Silver Spring, MD 20993, USA; sandra.fuentes@fda.hhs.gov (S.M.F.); eunhaebae@gmail.com (E.H.B.); subhikshananda@gmail.com (S.N.); dhanya.williams@fda.hhs.gov (D.K.W.); 2 Current Address: Ross University School of Medicine, Miramar, FL 33027, USA; 3 Current Address: Marie-Josée and Henry R. Kravis Center for Molecular Oncology, Memorial Sloan Kettering Cancer Center, New York, NY 10065, USA; 4 Present Address: Office of Device Evaluation, Center for Devices and Radiological Health, U.S. Food and Drug Administration, Silver Spring, MD 20993, USA

**Keywords:** simian foamy virus, spumaretrovirus, serotype, high-throughput sequencing, replication kinetics, cytopathic effect, reverse transcriptase activity

## Abstract

African green monkey (AGM) spumaretroviruses have been less well-studied than other simian foamy viruses (SFVs). We report the biological and genomic characterization of SFVcae_FV2014, which was the first foamy virus isolated from an African green monkey (AGM) and was found to be serotype 3. Infectivity studies in various cell lines from different species (mouse, dog, rhesus monkey, AGM, and human) indicated that like other SFVs, SFVcae_FV2014 had broad species and cell tropism, and in vitro cell culture infection resulted in cytopathic effect (CPE). In *Mus dunni* (a wild mouse fibroblast cell line), MDCK (Madin-Darby canine kidney cell line), FRhK-4 (a fetal rhesus kidney cell line), and MRC-5 (a human fetal lung cell line), SFVcae_FV2014 infection was productive resulting in CPE, and had delayed or similar replication kinetics compared with SFVmcy_FV21 and SFVmcy_FV34[RF], which are two Taiwanese macaque isolates, designated as serotypes 1 and 2, respectively. However, in Vero (AGM kidney cell line) and A549 (a human lung carcinoma cell line), the replication kinetics of SFVcae_FV2014 and the SFVmcy viruses were discordant: In Vero, SFVcae_FV2014 showed rapid replication kinetics and extensive CPE, and a persistent infection was seen in A549, with delayed, low CPE, which did not progress even upon extended culture (day 55). Nucleotide sequence analysis of the assembled SFVcae_FV2014 genome, obtained by high-throughput sequencing, indicated an overall 80–90% nucleotide sequence identity with SFVcae_LK3, the only available full-length genome sequence of an AGM SFV, and was distinct phylogenetically from other AGM spumaretroviruses, corroborating previous results based on analysis of partial *env* sequences. Our study confirmed that SFVcae_FV2014 and SFVcae_LK3 are genetically distinct AGM foamy virus (FV) isolates. Furthermore, comparative infectivity studies of SFVcae_FV2014 and SFVmcy isolates showed that although SFVs have a wide host range and cell tropism, regulation of virus replication is complex and depends on the virus strain and cell-specific factors.

## 1. Introduction

Early isolates of simian foamy viruses (SFVs), which belong to the recently described genus *Simiispumavirus* in the subfamily *Spumaretrovirinae* and family *Retroviridae* [1], were distinguished based on serotyping using neutralization assays. SFVcae_FV2014, previously known as SFV strain FV2014, isolated from the kidney tissue of an African green monkey (*Chlorocebus aethiops*), was designated as Type III [2] because it was serotypically distinct from other simian spumaretroviruses available at that time, which were SFV Type I and Type II, isolated from Taiwanese macaques (*Macaca cyclopis*) [3,4]. Subsequently, SFV strain LK3 was isolated from a lymphoblastoid cell line, established from a lymph node of a healthy *C. aethiops* [5] and designated as SFV serotype 3 based on sequence relatedness in hybridization experiments to the SFV Type III isolate FV2014 [6]. Unlike spumaretroviruses from macaque species, the number of reported foamy virus isolates from African green monkey (AGMs) are limited to FV2014 and LK3 [1]. The full-length sequence of SFV-3 strain LK-3 (referred to as SFV-3; [7]) was found to be more similar to SFV Type I than to the human foamy virus (subsequently found to be of chimpanzee origin [8]; current designation SFVpsc_huHSRV.13). Later, phylogenetic analysis based on short fragments in *env* obtained by DNA PCR amplification of 22 AGM spumaretrovirus sequences indicated diversity in AGM viruses and placed LK-3 and SFV-3/SFVcae_FV2014 on distinct branches of AGM spumaretroviruses [9]. It should be noted that in an earlier study [5], sera obtained from an SFVcae_LK3-infected AGM did not neutralize SFVcae_FV2014, and serum against the latter virus did not neutralize SFVcae_LK3. These results indicate that the serotype of SFVcae_LK3 needs to be further verified.

The high diversity of sequences in SFVs cocirculating with their host species is generally thought to be a result of intraspecies superinfection [10], and there is also evidence of interspecies virus infections and recombination. We have reported that the Taiwanese macaque isolate SFVmcy_2 (SFV serotype II, now referred to as SFVmcy_FV34[RF]), is a recombinant virus, which contains sequences in the *env*/SU region similar to SFVcae_LK3 (referred to as SFVagm-3; [11]). Additionally, Blochmann et al. reported isolation of SFVmac-R289hybAGM (currently designated as SFVmmu-R289hybAGM[RF]) from a rhesus macaque, which also had a similar recombination in *env* involving an AGM spumaretrovirus [12]. Since SFVs cocirculate and cospeciate with their hosts and are generally located in distinct geographical regions [8], foamy virus (FV) transmission and recombination may occur due to exposure of different NHP species in holding facilities during importation or handling in biomedical research facilitates and in zoos. Furthermore, human infections with FVs from various NHP species, including macaques and AGMs, have occurred due to occupational exposure in North America and in natural settings in Africa and Asia [8].

To study viral interactions of spumaretroviruses from different monkey species and potential for cross-species human infections, our laboratory previously developed well-characterized virus stocks of SFVs isolated from macaques [13,14]. In this study we describe the preparation and characterization of an SFVcae_FV2014 virus stock. Molecular and biological analysis showed that SFVcae_FV2014 is phylogenetically distinct from the AGM isolate SFVcae_LK3; furthermore, SFVcae_FV2014 had distinct replication kinetics from the macaque viruses, SFVmcy_FV21 and SFVmcy_FV34, based on infectivity studies using a variety of cell lines from different species.

## 2. Materials and Methods

### 2.1. Virus Preparation

SFV type 3 strain FV2014, an isolate from an African green monkey (grivet monkey kidney) [2], was obtained from American Type Culture Collection (ATCC, Manassas, VA, USA; catalogue number VR-218, lot 3W, titer 10^4.25^ per 0.2 mL in A-72 (canine tumor) cells; 8 days). The virus passage history provided by ATCC (host cells × number of passages) was: rabbit kidney (RabK) × 4, grivet monkey kidney (Grivet MkK) × 3, RabK × 10, primary rabbit kidney (PrRabK) × 1, and A-72 canine tumor cell line × 2 (ATCC CRL-1542).

A laboratory virus stock was prepared in *Mus dunni* cells, which were previously found to be highly susceptible to SFV replication [14]. Infected cells were grown until the cell culture was terminated due to extensive cytopathic effect (CPE) at passage 4 (day 11 after infection). Supernatant was collected and clarified by low speed centrifugation (1200 rpm for 10 min at 4 °C; GH-3.8 Swinging Bucket Rotor, Allegra 6KR Centrifuge, Beckman Coulter Life Sciences, Indianapolis, IN, USA) prior to filtration (0.45-μm-pore-size test tube top filter units; Corning, Cambridge, MA, USA). Aliquots were prepared for storage at −80 °C. The 50% tissue culture infective dose (TCID_50_) of the SFVcae_FV2014 virus stock was determined in MRC-5 cells using ten-fold dilutions with read-out for CPE on day 14 [15].

SFVmcy_FV21 and SFVmcy_FV34[RF], which were originally isolated from *Macaca cyclopis* [3,4], were obtained from ATCC (catalogue number VR-276, FV21, lot 5 WE and catalogue number VR-277, FV-34, lot 5 WE, respectively). The passage history at ATCC (host cells × number of passages) for SFVmcy_FV21 was: PrRabK × 13, KB (subline of HeLa) × 1, LLC-MK2 (normal rhesus monkey kidney) × 7, KB × 1, normal rat kidney × 2, Hep2 × 3 and A-72 × 2; and for SFVmcy_FV34[RF] was: PrRabK × 13, KB × 2, normal rat kidney × 2, and A-72 × 2. Viruses were amplified with low passage (< 5 or about < 15 days) in the *M. dunni* cell line [14]. Virus stocks were prepared when extensive CPE occurred, and reverse transcriptase (RT) activity was determined using a modified single-tube fluorescent product-enhanced RT assay (STF-PERT) [16], described in Section 2.3. The virus titer (TCID_50_) was determined in MRC-5 cells at day 14: SFVmcy_FV21 virus stock was 10^5.5^ TCID_50_ per mL and SFVmcy_FV34[RF] was 10^5.03^ TCID_50_ per mL.

### 2.2. PCR Assays and Copy Number Standard

Detection of SFVmcy_FV21 and SFVmcy_FV34[RF] sequences was based on the previously described PCR assay using Set B outer and inner primer sets from the long terminal repeats (LTR) region [14]. The outer primer pair consisted of forward primer 5′-CAGTGAATTCCAGAATCTCTTC-3′ and reverse primer 5′-CACTTATCCCACTAGATGGTTC-3′, and the inner primer pair consisted of forward primer 5′-CCAGAATCTCTTCATACTAACTA-3′ and reverse primer 5′-GATGGTTCCCTAAGCAAGGC-3′. The PCR conditions were modified: 95 °C 3 min, 95 °C 3 min, 95 °C 1 min, 55 °C 1 min, and 72 °C 1 min, for 35 cycles with extension 72 °C 10 min and 4 °C hold.

For detection of SFVcae_FV2014 sequences, primers were selected from the LTR-gag region of the full-length genome (Genbank accession number MF582544) using Primer-Blast (NCBI, NLM, National Institutes of Health, Bethesda, MD, USA). The outer primers were designated as SFVcae-F3 (5’-TGTTTGAGTCTCTCCAGGCTT-3’ extending from nucleotide position 1365 to 1385) and SFVcae-R3 (5’-CCATCTGTCATGCGAAGTCC-3’; nucleotide position 1937 to 1918), which amplified a 573 bp fragment. The inner primers were designated as SFVcae-F2 (5’-TAATGGGCAATGGCAATGCTT-3’; nucleotide position 1452 to 1472) and SFVcae-R2 (5’-TCTCTGTGATTGGGTTGTCTAGC-3’; nucleotide position 1910 to 1888), which amplified a 459 bp fragment. The first amplification was performed in 100 µL volume using 3 U of the Taq DNA polymerase (Roche Applied Science, Mannheim, Germany, catalogue number 11647679001) with 0.5 µM final concentration of the outer primer set and 250 ng of total cell DNA (QIAamp DNA Blood Mini Kit, Qiagen, Germantown, MD, USA) or 2 ng cDNA, prepared using Superscript VILO cDNA synthesis kit (Life technologies, Cat no. 11754) and cleaned using Zymo Research’s DNA clean and concentrator kit (catalogue number D4013). The second amplification was performed using the inner primer set and 10 µL PCR product from the first amplification in a total volume of 100 µL. For both amplifications, the conditions for the PCR were: 95 °C for 1 min, 55 °C for 1 min, and 72 °C for 1 min for 35 cycles. Initial denaturation was done at 95 °C for 3 min and the final extension at 72 °C for 10 min. Specificity of the LTR-gag PCR assays was determined by testing the three SFV isolates: The LTR PCR assay detected both SFVmcy_FV21 and SFVmcy_FV34[RF], whereas the LTR-gag PCR assay was specific for detection of SFVcae_FV2014.

The SFVcae_FV2014 LTR-*gag* fragment amplified using the outer primer pair was cloned and a copy number standard was made by spiking ten-fold dilutions of the DNA, ranging in the background of 0.25 µg cellular human genomic DNA (Roche, catalogue number 1169111200). Limit of detection for the second amplification was 10-100 copies.

Gag primers used for confirmation of sequence variants (described in Section 2.4.1) were SFV-3-F-1592 (5’-GTGAAAGGAATTGTGTA-3’) and SFV-3-R-2425 (5’-GAAGATGATGCAATAGG-3’) (covering the region extending from nucleotide positions 1592-2425). PCR amplification was performed in a total volume of 50 µL containing 2 ng cDNA, 0.5 μM of each oligo, 0.8mM dNTP mixture, 1× TaKaRa Ex Taq Buffer, and 5 U of TaKaRa Ex Taq (Clontech, catalogue number RR001A). PCR conditions were: 98 °C 3 min, 98 °C 10 s, 58 °C 1 min, and 72 °C 1 min, for 35 cycles with extension 72 °C 10 min and 4 °C hold.

### 2.3. Infectivity Analysis

Replication kinetics of SFVcae_FV2014, SFVmcy_FV21, and SFVmcy_FV34[RF] were compared using cell lines originating from different species and tissues shown in Table 1.

Cells used in this study originated from cell banks used in our other SFV infectivity studies (unpublished; [14]). MDCK, MRC-5, and Vero were grown in Eagle’s minimum essential medium (EMEM) and *M. dunni*, FhRK-4, and A549 were grown in Dulbecco’s modified Eagle medium (DMEM). All media were supplemented with 10% fetal bovine serum (GE Healthcare Hyclone, Logan, Utah, catalogue number SH30071.03; heat inactivated 56 °C for 30 min), 100 U of penicillin per mL, 100 µg of streptomycin per mL (Quality Biological, Gaithersburg, MD, USA; catalogue number 120-095-721), and 2 mM l-glutamine (Quality Biological; catalogue number 118-084-721). In addition, EMEM was also supplemented with 1 mM sodium pyruvate (Quality Biological; catalogue number 116-079-721) and 0.1 mM minimum non-essential amino acids (MEM-NEAA, Quality Biologicals, catalogue number 116-078-721).

SFV infection was carried out according to our laboratory’s standard protocol. Cells were planted in 25 cm^2^ flasks 24 h prior to infection and virus (193 TCID_50_) was added at 50–70% cell confluence. The number of cells planted were: *M. dunni*, 250,000; MDCK, 333,000; Vero, FRhK-4, A549; and MRC-5, 500,000. The cell passage at time of infection was: A549, p86; Vero, p129; MRC-5, p29; *M. dunni*, p45; FRhK-4, p45; and MDCK, p65). The optimum cell numbers were determined for each cell line and a low multiplicity of infection (approximate 0.0002–0.00045) was used. This was determined based on the most sensitive cell line in order to obtain differences in the replication kinetics for all of the viruses in the different cell lines. Cells were transferred to 75 cm^2^ flasks upon reaching confluence and passaged every 3–4 days. Filtered supernatant (0.45-µm-pore-size test tube top filter units; Corning, Cambridge, MA, USA) was collected and stored at −80 °C at each passage. Cultures were terminated at 4+ CPE (>75% cell death) or at day 30 to day 55, in case of slow or no CPE. Virus replication was monitored by microscopic observation of CPE progression in the cell monolayer and by determining the RT activity in filtered supernatant using a modified STF-PERT assay [16], in which the RT and the PCR steps were done in two steps, instead of using the one-step published protocol due to the discontinuation of AmpliWax PCR Gem 50. The assay was performed as described previously except that the RT reaction was done in the first step and then the PCR reaction mix was added immediately for the second step in the assay. All the samples collected from infectivity studies in one cell line were tested in duplicate or triplicate in the modified STF-PERT assay to compare kinetics of virus replication. Uninfected cells were set-up in parallel as control.

### 2.4. Preparation of Viral Nucleic Acid, Sequencing, and Bioinformatics Analysis

#### 2.4.1. High-Throughput Sequencing

Virus was concentrated by ultracentrifugation (45,000 rpm for 90 min at 4 °C; Rotor TLA-45, Beckman Coulter Optima MAX-XP Ultracentrifuge) or by using a Nanosep 30KD Omega Device (P/N OD030C33) with centrifugation at 5000× *g* for 20 min 4 °C. RNA was extracted using QIAamp Viral RNA Mini Kit (catalogue number 52904).

Viral nucleic acid prepared from SFVcae_FV2014 virus stock was sequenced using the MiSeq Illumina platform (CD Genomics, Shirley, NY, USA) as previously described [17], and a consensus virus sequence (SFVcae_FV2014 GenBank accession number MF582544) was generated by mapping the raw reads to the SFVcae_LK3 full-length genome as reference [7] (NCBI RefSeq accession number M74895). Default parameters were used for mapping: the length fraction (the minimum percentage of the total alignment length that must match the reference sequence at the selected similarity fraction) was set to be 0.5, and the similarity fraction (the minimum percentage identity between the aligned region of the read and the reference sequence) was set to be 0.8. The long terminal repeats (LTRs) were mapped separately using the same default mapping parameters to generate a complete and full-length consensus genome sequence of SFVcae_FV2014. Sequences in low-coverage regions were confirmed by virus-specific PCR assays. Open reading frames were identified using ORF Finder (https://www.ncbi.nlm.nih.gov/orffinder/).

Viral nucleic acid was prepared from filtered supernatant of SFVcae_FV2014-infected Vero cells at culture termination (day 15) for high-throughput sequencing using Illumina Hi-Seq (FDA/CBER Core Lab). The total numbers of quality, paired-end reads were 342,517,062 and the average read length was 99.2 bases. Sequence analysis was performed using CLC Genomics Workbench software, version 10.1.1 (CLC bio, Aarhus, Denmark). The sequence from SFVcae_FV2014 (accession number MF582544) was used as reference for the mapping. The default parameters were used, with the exception of length fraction and similarity fraction, which were set at 0.8 and 0.9, respectively.

To find virus variants, the reads were remapped using the SFVcae_FV2014 consensus sequence as the reference genome. Fixed ploidy variant detection analysis was done using the default setting with the noise threshold set at 10% and variant probability cutoff ≥35%, based on the known error rate of the Illumina sequencing platform [18]. Unaligned tail analysis helped to identify the previously reported splicing events in *tas*, *bet,* and *env* regions [19,20]. Ambiguous positions and splice sites were confirmed by PCR amplification and Sanger sequencing of gel-purified fragments (QIAquick gel extraction kit; Qiagen, catalogue number 28714).

#### 2.4.2. Sanger Sequencing

Sanger sequencing was used to confirm virus infection and HTS sequence results. Viral nucleic acid was extracted from SFVcae_FV2014 virus stock and cDNA synthesis done as described in Section 2.4.1. Total DNA was extracted from virus supernatant to confirm virus infection by PCR amplifications as described in Section 2.2. PCR fragments were analyzed on a 0.6% agarose gel and the expected size fragment was extracted and purified using QIAquick gel extraction kit (Qiagen, catalogue number 28714) for Sanger sequencing (CBER Core Facility).

### 2.5. Sequence Comparison and Phylogenetic Analysis

Comparative nucleotide sequence analyses and amino acid analyses were done for full-length, individual viral genes, and LTR with MegAlign (DNASTAR Lasergene, Inc. Madison, WI, USA) using the ClustalW method. Accession numbers for SFV sequences used in the analysis are shown in Table 2.

To compare the amino acid sequence of the SFVcae_FV2014 Gag at nucleotide position 1857 with the Gag of other foamy viruses, the Gag amino acid sequences from SFVcae_LK3, SFVmmu_K3T, SFVmcy_FV21, SFVmcy_FV34[RF], SFVpsc_huHRSV.13, EFVeca_1, FFVfca_FUV7, and SFVcae_FV2014 (accession numbers in Table 2) were aligned using ClustalW (MegAlign Pro DNASTAR Lasergene v.15).

Phylogenetic trees were generated based upon the nucleotide sequences in the entire *env* gene and in the SU region of *env* using MEGA7.0.14 (Molecular Evolutionary Genetics Analysis; www.megasoftware.net) as previously described [11]. The list of viruses and accession numbers used in the analysis is shown in Table 2. Briefly, nucleotide sequences were aligned in MEGA using ClustalW. The maximum-likelihood method based on the general time reversible model was chosen because it had the lowest Bayesian information criteria score in a model test performed in MEGA. The bootstrap consensus tree inferred from 1000 replicates was taken to represent the evolutionary history of the taxa analyzed. Branches corresponding to partitions reproduced in less than 50% bootstrap replicates are collapsed. The initial tree for the heuristic search was obtained automatically by applying neighbor-joining and BioNJ algorithms to a matrix of pairwise distances estimated using the maximum-composite-likelihood (MCL) approach and then selecting the topology with the superior log likelihood value. A discrete gamma distribution was used to model evolutionary rate differences among sites. All positions containing gaps and missing data were eliminated.

## 3. Results

### 3.1. Characterization of SFVcae_FV2014 Virus Stock

A virus stock of SFVcae_FV2014 was prepared and characterized for molecular and biological studies. The original virus from ATCC was amplified, similar to our other laboratory stocks of SFVs, in *M. dunni* with low passage (<5) to avoid nucleotide sequence changes that could potentially occur due to extended virus passage. *M. dunni* cells were used for virus amplification since these were previously found to produce a large number of extracellular virus particles compared to other cell lines [14]. Virus titer was 10^4.5^ per mL in MRC-5 cells [21].

**Figure 1:**
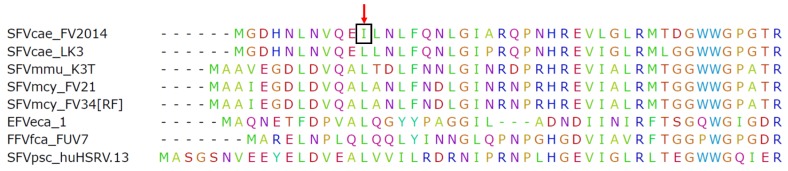
SFVcae_FV2014 variant. Amino acid sequence alignment for N-terminal Gag protein amino acids. The Gag sequences from different foamy viruses (FVs) were aligned using ClustalW. The first 50 amino acids of the alignment are shown. The red arrow points to the position of the conserved leucine in the CC1 domain of Gag; the change to isoleucine is indicated in the box.

Illumina MiSeq was used to determine viral sequences in the SFVcae_FV2014 virus stock. The consensus sequence was published [17]. Further analysis indicated the presence of a variant in *gag* at nucleotide position 1857: the variant frequency of A was 52% (represented in the consensus sequence) and T was 48%. This result was confirmed by Sanger sequencing. Interestingly, further passage of virus by inoculating *M. dunni* cells with the SFVcae_FV2014 virus stock indicated increased frequency of the T variant (82%) at culture termination, which corresponded to extensive CPE and peak RT activity. The one nucleotide change resulted in a conservative amino acid mutation from isoleucine to leucine in the consensus sequence of SFVcae_FV2014 located in the coiled-coil CC1 domain of the Gag protein. It is noted that the leucine residue is highly conserved in spumaretroviruses (Figure 1).

The certificate of analysis for the original ATCC stock indicated that it was contaminated with *Mycoplasma hominis*. However, the bioinformatics analysis of the MiSeq data for our laboratory stock prepared in *M. dunni* did not show sequences mapping to *M. hominis* (NCBI accession number NC_013511; data not shown), thus indicating the absence of mycoplasma contamination in the SFVcae_FV2014 virus used in our infectivity studies.

### 3.2. Studies of SFVcae_FV2014 Replication

**Figure 2:**
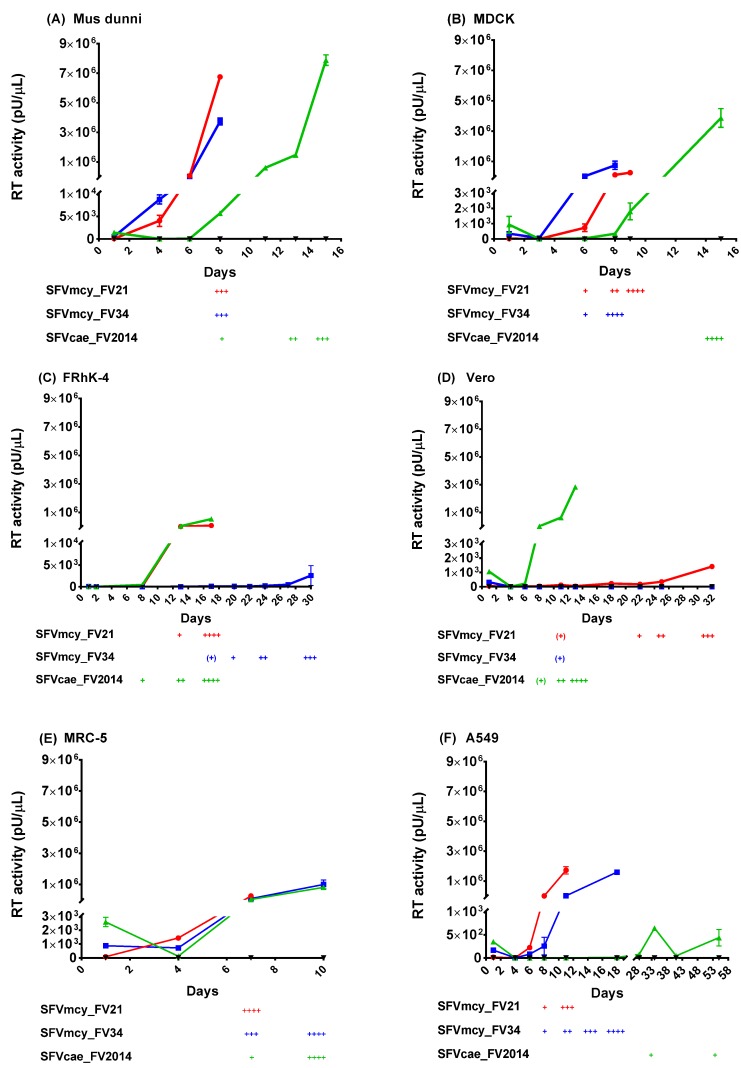
Kinetics of SFV replication. The kinetics of virus replication for SFVcae_FV2014 (▲) were compared with SFVmcy_FV21 (●) and SFVmcy_FV34 (■) in various cell lines from different species (**A**) *M. dunni*; (**B**) MDCK; (**C**) FRhK-4; (**D**) Vero; (**E**) MRC-5; and (**F**) A459 cells. Uninfected cells were the negative control (▼). Data from one of two independent studies are shown. Virus replication was determined based upon virus production in cell-free supernatant using the PERT assay (reported as pU/μL RT activity) and by visualization of cytopathic effect (CPE) development in the cells (reported as: + for up to 25% cell monolayer affected; ++, up to 50% monolayer affected; +++; up to 75% affected cells; and ++++, > 75% of cell monolayer affected). SFVmcy_FV21 infection in *M. dunni* was terminated at 3+ CPE due to insufficient cells for further passage. For comparison, the reverse transcriptase (RT) activity shown in the segmented Y-axis of the graph has the same Y-maximum value, although different cell lines had varied peak RT. The segmented, linear Y-axis shows the virus input and low-level RT activity on the bottom segment and the peak RT activity on the top segment. Error bars represent the SEM of virus supernatant samples tested in triplicate (*M. dunni*, MDCK, and MRC-5) or duplicate (Vero, FRhK-4, and A549) in the PERT assay. All samples from one cell line were tested in the same PERT assay.

Infectivity studies were done to compare the biological properties of SFVcae_FV2014 and SFV macaque isolates, SFVmcy_FV21 and SFVmcy_FV34[RF]. To remove differences due to previous propagation of the viruses in different host species, all the virus stocks were generated by low-passage in *M. dunni* cells. The kinetics of virus replication were determined using a low virus amount (193 TCID_50_), which was previously determined to allow virus propagation without early termination by extensive CPE. Cell lines from different host species and cell types were included to determine the influence of host on kinetics of SFV replication. Virus replication was evaluated based on development and progression of CPE in the cells and the increase in the RT activity produced in cell-free culture supernatant. The results from one of two studies are shown in Figure 2 (panels A–F): similar replication kinetics were seen in both.

In *M. dunni* (Figure 2A), SFVmcy_FV21 and SFVmcy_FV34[RF] showed similar and rapid replication: RT activity was initially seen in both SFVmcy isolates at day 4, which increased significantly (about 1000-fold) at day 8, resulting in culture termination due to extensive CPE. A delay in replication kinetics was seen with SFVcae_FV2014, where the initial RT activity and CPE was detected at day 8, which increased to a high level by day 15, and the culture was terminated due to extensive CPE. However, a similar level of RT activity was seen for the three viruses at culture termination (4–8 × 10^6^ pU per µL). In MDCK cells, SFV viruses had similar kinetics as in *M. dunni* except virus production was about a log lower for SFVmcy viruses (Figure 2B).

Notable differences in replication of SFVmcy and SFVcae viruses were seen in the simian cell lines. In the FRhK-4 cells (Figure 2C), SFVmcy_FV21 and SFVcae_FV2014 had similar kinetics of replication with initial RT detection at day 8 and culture termination due to extensive CPE on day 17, with about similar levels of RT activity (1–5 × 10^5^ pU per µL). Interestingly, SFVmcy_FV34[RF] had greatly delayed kinetics of replication: A very low level of RT activity was detected on day 8, which increased slowly but remained significantly low even at time of culture termination (about 75-fold lower); furthermore, there was also a delay in CPE progression, which was initially detected on day 17 but did not progress to extensive cell lysis even at day 30, when the experiment was terminated. In Vero cells (Figure 2D), the kinetics of replication for SFVcae_FV2014 were similar to *M. dunni cells*: with initial RT activity seen at day 6, with a fairly rapid and high increase in RT activity at time of culture termination on day 13 (about 3 × 10^6^ pU per µL). Unexpectedly, the replication of the SFVmcy viruses was significantly delayed compared to SFVcae, with low RT and slow CPE progression: In the case of SFVmcy_FV21, RT activity could be initially detected on day 6 and progressed slowly, and was detected above input background on day 11, increasing at a low level until culture termination on day 32, with RT activity < 2 × 10^3^ pU per µL. RT was not detected with infection of Vero using SFVmcy_FV34[RF]; although CPE was seen starting at day 11, no progression was seen during the culture period ending on day 32, at the time of experiment termination.

All three SFVs had rapid replication kinetics in MRC-5 cells (Figure 2E) although SFVmcy_FV21 had earlier culture termination at day 7 with 3- to 4-fold less RT activity compared with SFVmcy_FV34[RF] and SFVcae_FV2014, which had similar replication kinetics with culture termination on day 10 with high RT activity (about 10^6^ pU per µL). SFV infection of A549 cells (Figure 2F) showed differences in replication kinetics between the different viruses: SFVmcy_FV21-induced CPE progressed more rapidly with culture termination on day 11, whereas CPE progressed slower with SFVmcy_FV34[RF] with culture termination on day 18, although for both viruses, CPE was seen at day 8 and the RT activity was initially detected on day 6. Very slow kinetics of replication were seen with SFVcae_FV2014: a low-level RT activity was seen at day 29 with a low peak on day 34, which decreased on day 41, the next time point tested, and again increased on day 55, at culture termination, and the RT activity above input was seen only at day 34 and also at day 55, when the experiment was terminated; low CPE was seen at day 39 and thereafter, but without progression.

**Figure 3:**
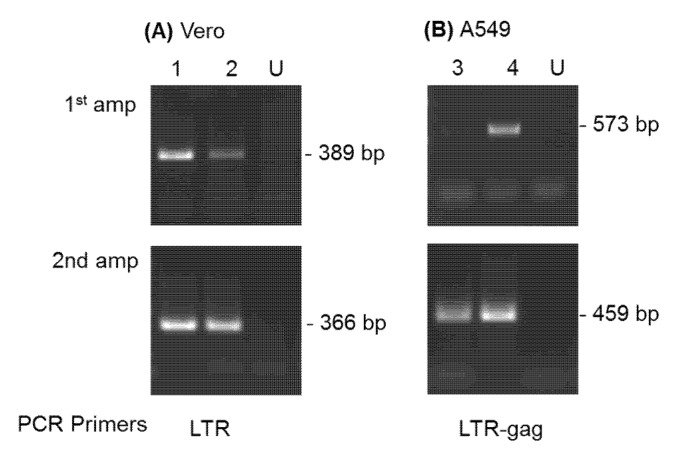
Detection of SFV sequences by DNA PCR analysis. DNA prepared at day 34 from SFVmcy_FV21- and SFVmcy_FV34[RF]-inoculated Vero cells (panel **A**, lanes 1 and 2, respectively) was analyzed using LTR primer sets (outer for 1st amplification and inner for second amplification). DNAs prepared from SFVcae-FV2014-inoculated A549 cells at day 34 and day 55 (panel **B**; lanes 3 and 4, respectively) were analyzed using LTR-gag outer and inner primer sets. DNA from uninoculated cells was obtained from each cell line (day 34) and included as negative control (lanes U).

Virus infection in Vero cells was confirmed for SFVmcy_FV21 and SFVmcy_FV34[RF] by PCR amplification of DNA prepared from cells collected on day 34 (Figure 3A, lane 1 and 2, respectively). Specific virus detection was verified by a second round of PCR amplification using internal primers, and virus identity was confirmed by nucleotide sequence analysis of the fragments from the first PCR amplification assays.

Virus infection in A549 cells was investigated by DNA PCR analysis of cells collected at day 34 and day 55 (Figure 3B, lanes 3 and 4, respectively). The results indicated an increase in SFVcae_FV2014 sequences with passage due to detection in the first round of PCR at day 55 but only in the second amplification with the day 34 sample. Virus identity was confirmed by nucleotide sequence analysis of the fragment obtained in the first PCR amplification at day 55.

### 3.3. SFVcae_FV2014 Genome Analysis

#### 3.3.1. Structure and Sequence Comparison

The genomic organization of the assembled SFVcae_FV2014 genome [17] was found to be similar to other SFVs: It had the expected structural genes (*gag*, *pol*, and *env*) and accessory (*tas* and *bet*) genes, an internal promoter, and long terminal repeats (LTRs) [8]. An 18-bp primer binding site (PBS) was identified at position 1713 to utilize the tRNA^Lys1,2^ isoacceptor for initiation of minus-strand DNA synthesis of spumaviruses [22]. The complete genomic sequence of SFVcae_FV2014 with alignment to the SFVcae_LK3 sequence is shown in Figure S1 and summarized in Table 3. The size of the *gag* and *pol* regions in SFVcae_FV2014 were the same as those in SFVcae_LK3, encoding 643 amino acid Gag and 1143 amino acid Pol proteins, respectively. There was also a 52 bp overlap between *gag* and *pol* and between *pol* and *env* in both viruses. However, there were differences in the size of *env*, *tas*, and *bet*, which resulted in a difference in size of encoded proteins between SFVcae_FV2014 and SFVcae_LK3. The *env* encoded a protein containing four additional amino acids in SFVcae_FV2014, which did not affect the reading frame (986 aa). The *tas* region of SFVcae_FV2014 had an early stop codon resulting in a 296 aa protein as compared to 298 aa for the Tas in SFVcae_LK3. An insertion of nucleotide A in position 11703 resulted in SFVcae_FV2014 having a longer Bet (504 aa) compared to SFVcae_LK3 (469 aa) and to the SFVmcy_FV21 and SFVmcy_FV34[RF] (497 aa and 308 aa, respectively).

Comparative sequence analysis indicated that SFVcae_FV2014 and SFVcae_LK3 had an overall sequence identity of about 70% to 90%, with the highest identity in the LTR, *pol*, *tas,* and *bet* regions (Table 4). Interestingly, in *env* there was high nucleotide and amino acid sequence identity in LP and TM, but only about 71% nucleotide and 67% amino acid identity in the SU region. SFVcae_FV2014 sequences were also compared with SFVmcy_FV21 and recombinant viruses SFVmcy_FV34[RF] and SFVmmu_R289HybAGM, which were previously found to have 77% amino acid identity to SFVcae_LK3 in the SU region (designated as SFVagm-3 in previous study; [11]). An overall sequence identity of 50–85% was seen, with the lowest being in the LTR, *gag*, *tas,* and *bet* regions. In *env*, the lowest was observed in the SU region, however, interestingly, SFVmcy_FV21 had about 74% sequence identity with SFVcae_FV2014, which was slightly higher than that seen between the two SFVcae viruses. Similarity plot analysis (Simplot) and BootScan analysis in Simplot did not indicate recombination in sequences of SFVmcy_FV21 or SFVcae_FV2014 (data not shown).

To further analyze the relatedness of SFVcae_FV2014 in the SU region with other SFVs that are available only as a partial sequence, a BLASTN search of the GenBank nt/nr database was done. SFVcae_FV2014 had between 71–78% nucleotide sequence identity and between 68–81% amino acid similarity with the other SFV sequences from AGM (data not shown). Some of these (SFVcae_agm4, SFVcae_agm5, SFVcae_agm20, and SFVcae_agm24) were selected as representative sequences of clusters A–D [9] in the phylogenetic analysis discussed below.

#### 3.3.2. Phylogenetic Analysis

**Figure 4:**
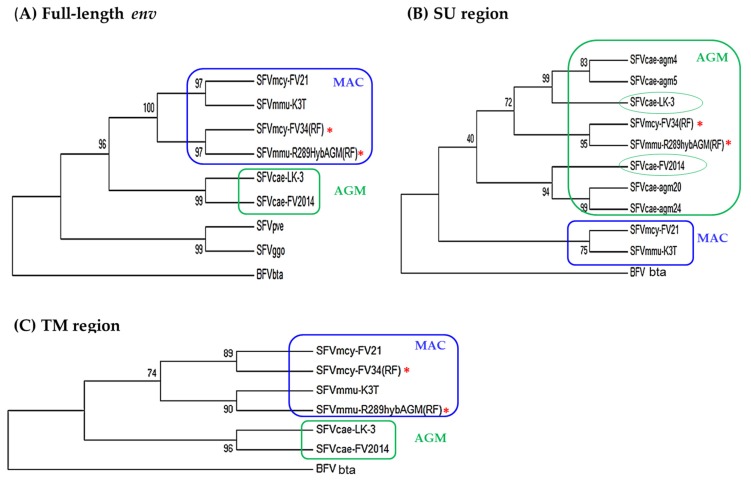
Phylogenetic analysis of SFV *env*. Results are shown for the nucleotide sequence in: (**A**) full-length *env*, using 2900 positions in the final dataset; (**B**) SU region using 1305 positions in the final dataset; and (**C**) TM region using 1242 positions in the final data set. The accession numbers of the virus sequences used for the analysis are shown in Table 2. BFVbta was used as the outgroup for all the trees. The percentage of replicate trees in which the associated taxa clustered together in the bootstrap test (1000 replicates) are shown next to the branches. Color indicates the clustering of AGM (green) and MAC (blue) isolates based on analysis of indicated regions in *env*. Recombinant virus is indicated with an asterisk.

Analysis of the evolutionary relatedness of the nucleotide sequences in LTR, *gag*, *pol*, *tas*, and *bet* showed that SFVcae_FV2014 and SFVcae_LK3 branched together and clustered with the macaque isolates, which included SFVmcy_FV21 and SFVmcy_FV34[RF] branched together, and the SFVmmu_R289HybAGM[RF] on a separate branch (data not shown). To evaluate the differences seen in SFV sequence identity in the LP, SU, and TM regions of *env* (Table 3), phylogenetic analyses were done in the full-length *env* and its subregions. This included SFVmmu_K3T, a naturally-occurring rhesus macaque virus isolated in our laboratory and in the SU region, also sequences from other AGM SFVs (SFVcae_agm4, SFVcae_agm5, SFVcae_agm20, and SFVcae_agm24). Constructed trees of the full-length *env*, SU, and TM regions are shown in Figure 4; results in the LP are not shown since they were similar to the TM. Analysis in *env* indicated the AGM viruses SFVcae_LK3 and SFVcae_FV2014 were branched together and clustered with the macaque isolates where SFVmcy_FV21 and SFVmmu_K3T branched together and SFVmcy_FV34[RF] and SFVmmu-R289HybAGM[RF] branched together. A difference in branch results was seen when analysis was done in the *env* subregions. In TM, SFVs branched with their monkey species: SFVmcy and SFVmmu were clustered on different branches and SFVcae isolates branched together. Similar results were seen in LP. However, in the SU region, although all of the SFVs from AGM were clustered together and the SFVs from macaques were clustered together, SFVcae-LK3 and SFVcae_FV2014 did not branch together and were grouped separately, along with different SFVcae_agm sequences: SFVcae_agm4 and SFVcae_agm6 with SFVcae_LK3 and SFVcae_agm20 and SFVcae_24 with SFVcae_FV2014 [9]. Interestingly, SFVmcy_FV34[RF] and SFVmmu-R289HybAGM[RF] branched together in a third group in the AGM cluster, further corroborating recombination in their SU region with SFV sequences from AGM [11,12].

## 4. Discussion

Previous analysis of spumaretroviruses in AGMs has focused on investigating intraspecies genetic diversity based on sequence analysis in the env/SU region [9]. The results showed prevalence of SFV strains that were divided into two phylogenetically diverged groups, each comprising distinct clusters (group 1 contained clusters A and B; group 2 contained clusters C and D). Furthermore, SFVcae_LK3 (designated as SFV-3/LK-3 in the reference paper) belonged to group 1 but was distinct from sequences in clusters A and B, whereas SFVcae_FV2014 (designated as SFV-3 in the reference paper) was an outlier and was divergent from both groups 1 and 2. In the present study we obtained the full-length genomic sequence of SFVcae_FV2014 and showed that it is a genetically distinct virus from SFVcae_LK3. The sequence differences between these SFV isolates might be due to distinct FV strains circulating in the AGMs in different geographical regions [23,24]. Although specific information about monkey origin is not available, it is reported that SFVcae_FV2014 was isolated from a grivet monkey kept in New York, USA [2], whereas other FV sequences in groups 1 and 2 were obtained from AGMs caught in the wild in Kenya and singly-housed in Freiburg, Germany [9]. Furthermore, SFVcae_LK3 was isolated from an AGM housed in Freiburg, Germany, but exposed to other AGMs and rhesus macaques during captivity [5]. Additionally, it is noted that SFVcae_LK3 was isolated from lymphoblastoid cells whereas the other sequences in groups 1 and 2 were PCR-amplified from kidney tissue. The phylogenetic differences in AGM SFV sequences from naturally-occurring viruses and the laboratory-isolates highlight the need for evaluating the biological properties of natural SFV isolates along with laboratory strains for developing relevant in vitro models to investigate SFV replication in NHPs and their potential for intra-, inter-, and cross-species virus transmission.

The large number of studies demonstrating broad distribution of SFVs in Old World and New World NHPs and species-specific prevalence of SFV strains have been based on sequence analysis of genomic regions [8,24,25,26,27,28,29,30,31,32,33,34]. Whole-genome analysis of some isolates has shown that genetically-diverse SFV strains circulating in different NHP species have contributed to the generation of recombinant viruses involving the SU region in *env* [11,12,35]. It is noted that sequence variation in SU was initially characterized in feline foamy viruses [36]. However, there is a lack of information regarding the biological properties of SFV strains that could be potential parent sequences involved in generation of recombinant viruses. We previously identified that SFVmcy_FV34[RF], isolated from *M. cyclopis*, was a recombinant virus with >90% overall nucleotide sequence identity to SFVmcy_FV21 (also isolated from the same monkey species), except in the SU region, which had about 76% nucleotide sequence identity to SFVcae_LK3, an AGM isolate [11]. Since recombination between viruses depends on the ability of two viruses to superinfect or coinfect the same species and replicate in the same cell type, we have investigated the biological properties of SFVmcy_FV34[RF] and its potential parent viruses SFVmcy_FV21 and SFVcae_FV2014, which had overall 80–90% nucleotide sequence identity to SFVcae_LK3. Cell lines from a range of different host species, tissues, and cell types were used to evaluate the host range and replication kinetics. To minimize variability in the virus preparations, the virus stocks were prepared in a similar manner and infectious titer obtained using the same assay. A low virus titer was used for infection to differentiate replication kinetics. As expected, there was productive infection with CPE in the fibroblastic cell lines [37,38], although higher virus production (RT activity) was seen in *M. dunni* [14] than in MRC-5. Furthermore, SFVcae_FV2014 had slightly slower replication kinetics in *M. dunni* compared with the SFVmcy viruses, whereas no difference was seen in MRC-5. Since all three viruses in the study were isolated from kidney tissue, kidney epithelial cell lines from rhesus monkey, AGM, and dog were included. Different kinetics of virus replication were seen: In MDCK cells, all three SFVs had rapid replication with CPE; in FRhK-4, SFVcae_FV2014 and SFVmcy_FV21 had similar rapid kinetics with CPE, whereas SFVmcy_FV34 had delayed kinetics ending in only 3+ CPE at day 30; in Vero only SFVcae_FV2014 showed rapid replication with CPE whereas SFVmcy_FV21 had delayed replication kinetics (3+ CPE at day 32) and SFVmcy_FV34 had a persistent infection without CPE progression (confirmed by PCR) until the culture was terminated on day 32. It should be noted that in our earlier study in Vero, SFVmcy_FV34 (then called SFV-2) showed low RT activity around day 30 but the lot of virus and infection titer used was different [14]. In the human lung carcinoma A549 epithelial cells, both SFVmcy viruses had productive infections with CPE, however, SFVcae_FV2014 had a persistent infection without CPE progression even on long-term culture (>50 days), although break-through low level RT activity was seen on day 33 and 55. It was noted that SFVcae_FV2014, SFVmcy_FV21, and SFVmcy_FV34[RF] had broad host ranges and infected all of the cell lines in the study, however, each had distinct replication kinetics except in MRC-5. This may be due to the MRC-5 cells being a diploid cell line whereas the others were continuous cell lines or because the viruses were titered in MRC-5. Further studies are needed to evaluate FV replication in primary tissues and cell cultures, diploid cells, and continuous cell lines to determine the relevant in vitro infectivity model that can accurately reflect in vivo infection.

The infectivity results in this study confirmed SFVs have a broad host range and further showed that regulation of virus replication post-entry is complex, depending on specific virus–host interactions. These results emphasize the need to study the biological properties of different SFV strains within a species to investigate their potential for replication and recombination as well as intra- and cross-species transmission. It is noted that a limitation of the current study is using SFVs that have an in vitro passage history in cell lines of different host species and cell types, however, FV genomes seem to be relatively stable and are therefore not expected to mutate in vitro or in vivo at a high rate like other retroviruses [9]. We are further investigating potential for SFV mutations in vitro by sequence analysis of viral genomes in productive and chronic infections.

Rapid replication of SFVcae_FV2014 was seen in Vero, however, a persistent infection was seen with SFVmcy_FV34[RF]. Since the SU region in the latter is related to the AGM viruses, the results suggest that sequences outside the SU may be involved in regulation of virus replication. We are currently investigating virus–host interactions to determine factors that could be important determinants of virus replication and virus latency. The overall results from the infectivity studies suggest a complex mechanism of regulation of SFV replication.

Transcription of SFVmcy_FV21 and SFVcae_LK3 (previously designated as SFV-1 and SFV-3) has been shown to be cell-line dependent [39] and productive, persistent infection of hematopoietic cells by SFV psc_huHRSV.13 (previously designated as HFV) has been reported [37]. Latently infected cultures with SFV have been established from epithelial cells [40,41,42] and lymphoblastoid cells [43]. Furthermore, in vivo SFV infection is widespread throughout the animal but remains generally latent except in the oral tissues [44]. Furthermore, recent in vivo studies have confirmed in vivo replication in oropharyngeal tissues [45] and further identified that it is limited to the short-lived differentiated epithelial cells [46]. Our infectivity studies analyzing the replication kinetics of three SFVs in different cell lines from various species indicates that virus replication is dependent on the virus strain and interaction of virus-specific sequences with host-specific factors is an important determinant of outcome of infection. Studies are underway with naturally-occurring SFV strains from rhesus macaques to identify the virus–host interactions involved in SFV replication.

## Figures and Tables

**Table 1:** Cell lines used in infectivity studies.

Cell Name	Source	Host Species	Tissue Origin	Cell Type
*Mus dunni*	[14]	wild mouse	tail	fibroblast
MDCK (NBL-2)	ATCC, CCL-34	dog	kidney	epithelial
FRhK-4	ATCC, CRL-1688	rhesus macaque	fetal, kidney	epithelial
Vero	ATCC, CCL-81	African green	kidney	epithelial
		monkey		
MRC-5	ATCC, CCL-171	human	lung, fetal	fibroblast,
				diploid
A549	ATCC, CCL-185	human	lung, carcinoma	epithelial

**Table 2:** Simian foamy virus (SFV) isolates used for phylogenetic analysis and sequence alignments.

Virus	Previous Designation	Species of Virus Isolation	Accession Number ^1^
SFVmcy_FV21	SFVmcy-1, (SFV serotype I)	Taiwanese macaque	NC_010819
SFVmcy_FV34[RF]	SFVmcy-2, (SFV serotype II)	Taiwanese macaque	KF026286
SFVmmu_K3T	SFVmmu-K3T	Rhesus macaque	KF026288
SFVmmu_R289HybAGM[RF]	SFV-R289HybAGM	Rhesus macaque	JN801175
SFVcae_FV2014	SFV 3	African green monkey	MF582544
	(SFV serotype III)		
SFVcae_LK3	SFVagm-3	African green monkey	NC_010820
SFVcae_agm4	agm4	African green monkey	AJ244075
SFVcae_agm5	agm5	African green monkey	AJ244067
SFVcae_agm20	agm20	African green monkey	AJ244091
SFVcae_agm24	agm24	African green monkey	AJ244090
SFVpve	SFVcpz	Chimpanzee	NC_001364
SFVpsc_huHRSV.13	HFV	Chimpanzee	KX08159
SFVggo	SFVgor	Gorilla	HM245790
BFVbta	BFV	Cow	NC_001831
EFVeca_1	EFVeca	Equine	AF201902
FFVfca_FUV7	FFVfca	Feline	Y08851

^1^ GenBank or NCBI Reference Sequence.

**Table 3:** Comparison of genomic structures of SFVcae isolates.

Viral Regions	Location ^1^	SFVcae_FV2014 LTR/gene ^2^	ORF^ 3^	Location ^1^	SFVcae_LK3 LTR/gene ^2^	ORF ^3^
LTR	1–1710	1710		1–1708	1708	
*gag*	1827–3758	1932	643	1825–3756	1932	643
*pol*	3706–7137	3432	1143	3704–7135	3432	1143
*env*	7085–10045	2961	986	7083–10031	2949	982
*tas*	10,015–10,905	891	296	10,001–10,897	897	298
*bet*	10,015–10,285, 10,581–11,824	1515	504	10,001–10,271, 10,567–11,705	1410	469
LTR	11,418–13,127	1710		11,404–13,111	1708	

^1^ nucleotide position; ^2^ number of nucleotides; ^3^ number of amino acids.

**Table 4:** Sequence comparison of SFVcae_FV2014 and different SFV isolates.

SFV Isolates	% Sequence Identity									
		LTR	*gag*	*pol*	*env*	*env*-LP	*env-*SU	*env-*TM	*tas*	*bet*
SFVcae_LK3	nt	88.7^1^	81.5	86.3	79.1	79.6	71.1	86.6	90.2	88.8
	aa		85.4	91.6	81.5	86.5	67.3	93.9	91.3	88.5
										
SFVmcy_FV21	nt	66.9	68.1	81.7	75.0	71.4	73.8	77.0	66.1	61.8
	aa		65.8	86.1	78.2	73.0	73.5	84.8	52.2	51.4
SFVmcy_FV34(RF)	nt	65.8	68.8	81.9	75.0	72.2	69.1	76.6	65.4	64.4
	aa		66.7	86.3	78.2	73.8	63.2	83.9	52.5	50.9
										
SFVmmu_R289(RF)	nt	67.6	69.6	81.5	73.4	73.8	68.3	77.8	66.7	62.2
	aa		67.6	86.4	74.5	76.2	63.7	85.3	53.7	51.2

^1^ Numbers are percent identity using the ClustalW alignment option in MegAlign (DNASTAR Lasergene).

## Supplementary Materials

The following are available online at https://www.mdpi.com/1999-4915/12/4/403/s1, Figure S1. Nucleotide sequence alignment of SFVcae_FV2014 and SFVcae_LK3. Sequences were aligned using EMBOSS Stretcher version 6.6.0 using default parameters (http://www.ebi.ac.uk/Tools/psa/emboss_stretcher/help/index-nucleotide.html). Nucleotide positions are indicated. Vertical lines indicate identical bases and dots indicate different bases. The LTRs, *gag*, *pol*, *env*, *tas*, and *bet* regions are indicated. The TATA, poly A^+^ signal, and poly A^+^ sites are underlined in the LTRs. The 3 nucleotides for the start and termination codons of genes are shown in red. The TATA in tas is underlined and the start site of the internal promoter (IP) is indicated by an arrow. The regulatory signals are indicated based upon homology to the SFVcae_LK3 sequence [7].
