# Supplementary material for: Genome Analysis and Replication Studies of the African Green Monkey Simian Foamy Virus Serotype 3 Strain FV2014
Source: Viruses. 2020 Apr 6;12(4):403. doi: 10.3390/v12040403 (PMC7232438; doi:10.3390/v12040403)
Supplement: Supplementary file 1 [file viruses-12-00403-s001.zip › viruses-694850-supplementary.docx]

SFVcae_FV2014 1 TGTGGCTGACATCCACTAGAATGACAGGCCCCCAGGAAGATGATTATTGG 50

|||||||||.||||||||.|||...|||||||||||||.|.|||||||||

SFVcae_LK3 1 TGTGGCTGATATCCACTAAAATAGTAGGCCCCCAGGAAAAGGATTATTGG 50

SFVcae_FV2014 51 AAAGATGCCTATCGATGGGGATATTTCCCTTTGGTCCCAAATAAACATCA 100

|.|||.||.||.||.||||||||||||||.||.||.||.|||||.|||||

SFVcae_LK3 51 AGAGACGCATACCGTTGGGGATATTTCCCCTTAGTTCCCAATAAGCATCA 100

SFVcae_FV2014 101 TCCTGGGTGGACTAGATATTTAACTAAATTTAAGATCTCTAGATTCTCCA 150

||||||||||||||||.||||||||||.||.|||||.|||||||||.|||

SFVcae_LK3 101 TCCTGGGTGGACTAGACATTTAACTAAGTTCAAGATTTCTAGATTCGCCA 150

SFVcae_FV2014 151 CTCCTGCTGATATCCAAAAGATTACAGATGAACTTCTCCCTAGAGGAGCA 200

||||||||||.||||||||||||...||||||||||||||||||||||||

SFVcae_LK3 151 CTCCTGCTGACATCCAAAAGATTGTTGATGAACTTCTCCCTAGAGGAGCA 200

SFVcae_FV2014 201 AGCATTGTTACAGCTGATGGATCAAAGTATGAAAGCACTAGAAAAGTGCA 250

|||||||||||||||||||||...|.|||||||||||||||.||.||.||

SFVcae_LK3 201 AGCATTGTTACAGCTGATGGAAATAGGTATGAAAGCACTAGGAAGGTACA 250

SFVcae_FV2014 251 TTTGGTTAATGAAGGAACTCTCAAAGAATACCAAGACAAAAATAGAGAAA 300

|||.||||||||||||||.||..||||.||||||| |.||||||..||||

SFVcae_LK3 251 TTTAGTTAATGAAGGAACCCTTGAAGAGTACCAAG-CTAAAATAAGGAAA 299

SFVcae_FV2014 301 TAGAAGAAAAATATGGATGTGGATGTTGTCTGTCCTCAGATAGTGATGAT 350

|||||||||.|||||||||||||||||||||.||.|||||||||||||||

SFVcae_LK3 300 TAGAAGAAAGATATGGATGTGGATGTTGTCTTTCTTCAGATAGTGATGAT 349

SFVcae_FV2014 351 GATGATTATTCCGAGGATACACCTGATACAGAAAGTACTAGTGTTGAAGA 400

||.||||||||.||.|||||.|||||.||.||||.|||.|||||.||.||

SFVcae_LK3 350 GAGGATTATTCTGAAGATACCCCTGACACTGAAACTACAAGTGTAGAGGA 399

SFVcae_FV2014 401 AGACTAAAATGCAGGGTACAGTGTTGTTCATTTACATAATCTGCTTAGCA 450

|||.|||||..||||||||||||||.||.|||||||||||||||||||||

SFVcae_LK3 400 AGATTAAAACACAGGGTACAGTGTTATTTATTTACATAATCTGCTTAGCA 449

SFVcae_FV2014 451 ACTGCTTATGCAATAAGAGTGATTCAGTATATTGTTTAGGAATAAGATAT 500

|||||||||||.. ||||.|||||||||||||||||||||||||||||||

SFVcae_LK3 450 ACTGCTTATGCTC-AAGAATGATTCAGTATATTGTTTAGGAATAAGATAT 498

SFVcae_FV2014 501 AGTTTAATAGTAGTTAATCCTTAGGGAGTATTTGGTGGAAACGACTAAGT 550

||||||..||.|||||||||||||||||||||||||||||||||||.|||

SFVcae_LK3 499 AGTTTATAAGAAGTTAATCCTTAGGGAGTATTTGGTGGAAACGACTGAGT 548

SFVcae_FV2014 551 GACAC-AAGTTTATTCACCATACTCTCAATAGGAGCCACTAGTTGAGCCT 599

||||. ||||||||||||||||||||||||||||||||||||||||||||

SFVcae_LK3 549 GACATGAAGTTTATTCACCATACTCTCAATAGGAGCCACTAGTTGAGCCT 598

SFVcae_FV2014 600 GTGTGTTCAAATCCATGCTCAGCTAAAGTGACTCCCTTTTAGTTTCACTT 649

||||||||||||||||||||||||||||||||||||||||||||||||||

SFVcae_LK3 599 GTGTGTTCAAATCCATGCTCAGCTAAAGTGACTCCCTTTTAGTTTCACTT 648

SFVcae_FV2014 650 TTAGGTTAAG-ATAGATATAGATTCCATATAATCCTAAGGGAGTATGTGG 698

| |||.|||| |||| ||||..|..|||||||||||||||||||||||||

SFVcae_LK3 649 T-AGGATAAGTATAG-TATAAGTATCATATAATCCTAAGGGAGTATGTGG 696

SFVcae_FV2014 699 ACCTTCTTGTTAGGAATTAGTTTAAGATAGTCCACAGCTCCCTTCTTTTT 748

||||||||||||||||||||||||||||||||||||||||||||||||||

SFVcae_LK3 697 ACCTTCTTGTTAGGAATTAGTTTAAGATAGTCCACAGCTCCCTTCTTTTT 746

SFVcae_FV2014 749 AAGTTCAAGACTATGTATAGTTTGTTGGCTCATAACAGATAAAGTGCTCA 798

.|||||.||.||.|||..|||||||||||||||| |||||||||||||||

SFVcae_LK3 747 GAGTTCTAGTCTTTGTTAAGTTTGTTGGCTCATA-CAGATAAAGTGCTCA 795

SFVcae_FV2014 799 TAAGACAGGAAACCGCAACCGGGTAAAGGTTAGCACAGTTTGTTAAGCTA 848

|.|.|||||||||||||||||||||||||||||||||||...||||||||

SFVcae_LK3 796 TTAAACAGGAAACCGCAACCGGGTAAAGGTTAGCACAGTAAATTAAGCTA 845

SFVcae_FV2014 849 GTCGTTACCCAAGAGCCCGGTAAGCATTTAAGTGGTTCGAGTCTCTTTAA 898

|..|||||.|||||||||||||||||||.||||.||||||.||.||||||

SFVcae_LK3 846 GCAGTTACTCAAGAGCCCGGTAAGCATTCAAGTAGTTCGAATCCCTTTAA 895

SFVcae_FV2014 899 TGCTGACGGATTGCTCTTTAGTGAAGTGATGTAATATGTTTTTGTGAATC 948

||||||||||||||||||||||||.||||||||||.|||||||||.|...

SFVcae_LK3 896 TGCTGACGGATTGCTCTTTAGTGAGGTGATGTAATTTGTTTTTGTAATCT 945

SFVcae_FV2014 949 AAAATGTGTTTCTGAACAGGAAGTAAAACAAGAAAGGGAATGGCTAAACT 998

.||||||||.|.||.|.|||||||...||||.||||||||||||||||||

SFVcae_LK3 946 GAAATGTGTATTTGTATAGGAAGTTGTACAAAAAAGGGAATGGCTAAACT 995

SFVcae_FV2014 999 TGTTTAAGCTCAAGCAAACATTTAGCTCTTTCCTTTGCTTTTGGAGTTCG 1048

||||..|||||.||||||||||||||..|||||||||||||||.||||||

SFVcae_LK3 996 TGTTACAGCTCGAGCAAACATTTAGCAATTTCCTTTGCTTTTGAAGTTCG 1045

SFVcae_FV2014 1049 AGTCTTGTAATTGCATTTTGAGCACTGTATTTAGAATAACTTAAGTATGG 1098

|.|||||||||...|||||.|||||...|..||.|| |.|||||||||||

SFVcae_LK3 1046 AATCTTGTAATAATATTTTAAGCACATGAACTATAA-AGCTTAAGTATGG 1094

SFVcae_FV2014 1099 AAAAATCTCCAAGTATGAGTCACGAGATGCTTGGCTCACTGCGTTGGACG 1148

|||||||..||||||||||||||||||||.||||||||||||||||||||

SFVcae_LK3 1095 AAAAATCCTCAAGTATGAGTCACGAGATGTTTGGCTCACTGCGTTGGACG 1144

SFVcae_FV2014 1149 ACTGGATAGAAGCTTCAACAGTCGGGACAGCATCTCGAAGAAGGCCTCCA 1198

||||||.||||||||||||||||||||||||||||||||||||||||||.

SFVcae_LK3 1145 ACTGGAAAGAAGCTTCAACAGTCGGGACAGCATCTCGAAGAAGGCCTCCG 1194

SFVcae_FV2014 1199 GAGTGAAAGAGTGGAAAT-GAAATCTCCTCATTCAGAGTGCCTTCTTTTT 1247

||.|||||||||..|||| |||.|||||||||||||||.||||||||||.

SFVcae_LK3 1195 GAATGAAAGAGTAAAAATTGAAGTCTCCTCATTCAGAGAGCCTTCTTTTG 1244

SFVcae_FV2014 1248 -AACTTTAGGTAGAATATAGTTTCCAGTAGGATAAACTTTTGTACTAGCT 1296

||.||||||||||..|.||||||.|.|||.|||||||||||||..|||.

SFVcae_LK3 1245 GAATTTTAGGTAGAGAAAAGTTTCTAATAGAATAAACTTTTGTATCAGCA 1294

**U3|R**

SFVcae_FV2014 1297 GATAGATAGGATATATAATCTCTGCTTTAGATTGTACGAGAGCTCTTCAC 1346

||||||||||||||||||||.|||||||||||||||||.||||||..|||

SFVcae_LK3 1295 GATAGATAGGATATATAATCCCTGCTTTAGATTGTACGGGAGCTCACCAC 1344

SFVcae_FV2014 1347 TACTCGCTGCGTCGAGAGTGTTTGAGTCTCTCCAGGCTTGGTAAGATATA 1396

|.||||||||||||||||||||.|||||||||||||||||||||||||.|

SFVcae_LK3 1345 TGCTCGCTGCGTCGAGAGTGTTCGAGTCTCTCCAGGCTTGGTAAGATAGA 1394

SFVcae_FV2014 1397 AACTTTGGTATTCTCTGTATTATCTATGATCCAATAATACTCTGCTTATA 1446

| ||||||||||||.|||||| |.|||||||||||.||||||||||||||

SFVcae_LK3 1395 A-CTTTGGTATTCTATGTATT-TTTATGATCCAATTATACTCTGCTTATA 1442

SFVcae_FV2014 1447 GATTGTAATGGGCAATGGCAATGCTTAAT-AATTAATGAAGTTTATGGTG 1495

||||||||||||||||||||||||||.|| |||.|||||. |||||||||

SFVcae_LK3 1443 GATTGTAATGGGCAATGGCAATGCTTTATCAATGAATGAT-TTTATGGTG 1491

SFVcae_FV2014 1496 AATTAAGTTCATATATGTTTAAAGAAGTTTATCAATAAACCGACTTAATT 1545

|||||||||||||||||||||||||||||||.||||||||||||||||||

SFVcae_LK3 1492 AATTAAGTTCATATATGTTTAAAGAAGTTTAACAATAAACCGACTTAATT 1541

**R|U5**

SFVcae_FV2014 1546 CGAGAACCAGATTTATTAGTATTGTCTCTTTCTATACTTAA-GTAAAGTG 1594

|||||||||||||||||||||||||||||||||||||||.| |.||||||

SFVcae_LK3 1542 CGAGAACCAGATTTATTAGTATTGTCTCTTTCTATACTTTATGCAAAGTG 1591

SFVcae_FV2014 1595 AAAGGAATTGTGTATTAGCCTTGCTTAAGAAAATCATTTAACAGTATAAG 1644

||||||.||||.|||||||||||||||..|.|..|||.||...|||||||

SFVcae_LK3 1592 AAAGGAGTTGTATATTAGCCTTGCTTATAAGAGCCATCTAGTGGTATAAG 1641

SFVcae_FV2014 1645 TGTGTACTACACTTATCATAAGGGGTGATATT-TCTAAGGATAATCAATA 1693

|||..|.|||||||||||||||.||||..||| |.|||||||||||||||

SFVcae_LK3 1642 TGTAGATTACACTTATCATAAGAGGTGGAATTCTTTAAGGATAATCAATA 1691

**LTR| PBS**

SFVcae_FV2014 1694 CACAATAATCCATGACAATTGGCGCCCAACGTGGGGCTCGAATATAAGTC 1743

.||||.|.||||.|||||||||||||||||||||||||||||||||||||

SFVcae_LK3 1692 TACAAAATTCCACGACAATTGGCGCCCAACGTGGGGCTCGAATATAAGTC 1741

SFVcae_FV2014 1744 GGGTAATATTGTTTTAAGCCTTTTCAGG-CTAATATTATATCCCTAGGGA 1792

||||..||||.....|..|.|.||.||| ||.|.|.| ||||||||||||

SFVcae_LK3 1742 GGGTCTTATTAAAAAAGACTTATTTAGGTCTTACAAT-TATCCCTAGGGA 1790

**|*gag***

SFVcae_FV2014 1793 CCTTCACGCACGGCGGAAGGCATAAGCACTCAAA**ATG**GGTGATCATAACT 1842

|||||||||||.||||||||.||||||||||||||||||||||||.||||

SFVcae_LK3 1791 CCTTCACGCACTGCGGAAGGAATAAGCACTCAAA**ATG**GGTGATCACAACT 1840

SFVcae_FV2014 1843 TGAATGTTCAAGAAATACTGAACTTATTTCAAAATTTGGGAATTGCTAGA 1892

||||||||||||||.|..|.|||.|.||.||.|||.||||.|||.|.|||

SFVcae_LK3 1841 TGAATGTTCAAGAACTCTTAAACCTTTTCCAGAATCTGGGTATTCCCAGA 1890

SFVcae_FV2014 1893 CAACCCAATCACAGAGAAGTCTTAGGACTTCGCATGACAGATGGCTGGTG 1942

|||||.||.||.|||||||||.||||||||||.|||..||..||||||||

SFVcae_LK3 1891 CAACCAAACCATAGAGAAGTCATAGGACTTCGAATGTTAGGAGGCTGGTG 1940

SFVcae_FV2014 1943 GGGTCCTGGAACCCGATATAATTTAGTGTCAATTTTTCTACAAGATGATT 1992

||||||.||.|||||.||||.|.|.||.||.||.|||.||||||||||||

SFVcae_LK3 1941 GGGTCCAGGCACCCGTTATATTCTGGTTTCTATCTTTTTACAAGATGATT 1990

SFVcae_FV2014 1993 CTGGGCAACCTTTACAACAGCCCAGGTGGAGACCTGAAGGAAGACCAGTA 2042

||||.||||||||.|||||.||..|.||||||||.||.||.|||||.||.

SFVcae_LK3 1991 CTGGACAACCTTTGCAACAACCTCGATGGAGACCCGAGGGTAGACCTGTT 2040

SFVcae_FV2014 2043 AATCCTCTAGTGCATAATACTTTGGAAGCCCCCTGGGGGGACCTTAGATT 2092

||||||.||||.||.|||||..|.||||||||.||||||||..|.|||..

SFVcae_LK3 2041 AATCCTTTAGTACACAATACCATAGAAGCCCCTTGGGGGGAATTAAGACA 2090

SFVcae_FV2014 2093 AGCATTTGAGGATTTGGATGTAGCTGAAGGTACCCTTAGGTTGGGGCCTT 2142

|||||||||.||..|.|||||.||.|||||.||..|.|||||.||.|||.

SFVcae_LK3 2091 AGCATTTGAAGACCTAGATGTGGCGGAAGGAACTTTAAGGTTTGGTCCTC 2140

SFVcae_FV2014 2143 TAGCTAATGGTAATTGGATACCTGGAGATGAATATTCATTGGAATTTCAG 2192

||||||||||.||||||||.|||||||||||||||||..|||||||.||.

SFVcae_LK3 2141 TAGCTAATGGGAATTGGATTCCTGGAGATGAATATTCCATGGAATTCCAA 2190

SFVcae_FV2014 2193 CCTCCCTTAGCACAAGAAATTGCTCAAATGCAAAGAGATGAATTAGAAGA 2242

||.||.|||||.||.|||||||||||||||||.||.||||||||||||||

SFVcae_LK3 2191 CCCCCATTAGCCCAGGAAATTGCTCAAATGCAGAGGGATGAATTAGAAGA 2240

SFVcae_FV2014 2243 AATGTTGGATGTTATAGGACAAATTTGTGTACAAGTAATAGACCTAGTAG 2292

|||.||.|||.|.|.||||||||||||||.||||||.|||||..||||||

SFVcae_LK3 2241 AATTTTAGATATAACAGGACAAATTTGTGCACAAGTCATAGATTTAGTAG 2290

SFVcae_FV2014 2293 ATATGCAAGATGCTCAAATTAGAGGTTTTGAGAGACAATTGCAACAGAGA 2342

|||||||||||||.|||||.||.|||||.||.||||...|.|||.|.|||

SFVcae_LK3 2291 ATATGCAAGATGCACAAATCAGGGGTTTGGAAAGACGCATACAAGATAGA 2340

SFVcae_FV2014 2343 CAAGGTATTGGAGAAAACTTACCAGTTGCTGGAATACAAGCACCACCATC 2392

|.||||.|..|.||.||||||||||||||.||||||||||||||||||||

SFVcae_LK3 2341 CTAGGTTTGAGGGATAACTTACCAGTTGCCGGAATACAAGCACCACCATC 2390

SFVcae_FV2014 2393 TAGTCCAATTGGTCAGCCTATTGCATCATCTTCGCTACAGCCCCTTCCAG 2442

|||||||||||||||.|||||||||||||||||.||.||||||.||||||

SFVcae_LK3 2391 TAGTCCAATTGGTCAACCTATTGCATCATCTTCACTTCAGCCCATTCCAG 2440

SFVcae_FV2014 2443 GTGCTAGTTCATCACCTGCTGATTTAGGATGGGGTTCTGGAGCACCAGGC 2492

|..||||.||||||||.||.|||.||| |||||.| ||||| |||||.|.

SFVcae_LK3 2441 GATCTAGCTCATCACCAGCAGATCTAG-ATGGGAT-CTGGA-CACCAAGG 2487

SFVcae_FV2014 2493 AATATAGACCCTAGATTGTCTAGGGTAGCCTATAATCCATTTTTGCCAGG 2542

.|.||||||||||||||||||||||||||||||||.||.|||||||||||

SFVcae_LK3 2488 CAAATAGACCCTAGATTGTCTAGGGTAGCCTATAACCCCTTTTTGCCAGG 2537

SFVcae_FV2014 2543 ACCAAGTGATGGAACTGGAGGATCTATCCCAGTGCAGCCTAGTGCTCCAC 2592

|.|.||||||||..|.||.|||||.|||||.||.|||||.||||||||.|

SFVcae_LK3 2538 ATCTAGTGATGGGTCAGGGGGATCAATCCCGGTTCAGCCGAGTGCTCCTC 2587

SFVcae_FV2014 2593 CAGCGATATTTCCTTCTCCTCCCTCACTACCTGCTCCT------CAACCT 2636

|||||.|..||||.||....|||||.|||||||||||. ||.||.

SFVcae_LK3 2588 CAGCGGTTCTTCCATCCTTACCCTCGCTACCTGCTCCAGTGTCACAGCCA 2637

SFVcae_FV2014 2637 GTCATACAGTATGTGGCTCCACCTCCGGCTCCTGCTCAACAAGCTATACC 2686

.|.||.||||||||.||||.||||||.|.||||||||..||||||||.||

SFVcae_LK3 2638 ATAATTCAGTATGTTGCTCAACCTCCCGTTCCTGCTCCTCAAGCTATTCC 2687

SFVcae_FV2014 2687 AATTCAACATATTAGAGCTGTAACAGGCAACACACCCACTAATCCCAGAG 2736

.||||||||||||.||||.||.|||||.||||||||.||||||||.||||

SFVcae_LK3 2688 TATTCAACATATTCGAGCAGTGACAGGAAACACACCAACTAATCCAAGAG 2737

SFVcae_FV2014 2737 AAATACCTATGTGGCTTGGACGACATTCAGCTGCCATTGAAGGAGTTTTT 2786

|.||||||||||||||||||.||||.||.||.|||||.||||||||||||

SFVcae_LK3 2738 ACATACCTATGTGGCTTGGAAGACACTCGGCAGCCATAGAAGGAGTTTTT 2787

SFVcae_FV2014 2787 CCTATGACTACACCAGATCTAAGATGTCGTGTTGTCAATGCTCTGGTAGG 2836

||||||||||||||.|||||..|.||.||.||.|||||.||..|..||||

SFVcae_LK3 2788 CCTATGACTACACCTGATCTTCGTTGCCGAGTGGTCAACGCCTTAATAGG 2837

SFVcae_FV2014 2837 AGGTAGTCTTGGACTTTCATTGGAACCTATTCATTGTGTGAATTGGGCCG 2886

|||.||||||||||||||.||.|||||||||||||||||.||||||||.|

SFVcae_LK3 2838 AGGCAGTCTTGGACTTTCTTTAGAACCTATTCATTGTGTTAATTGGGCAG 2887

SFVcae_FV2014 2887 CAGTTGTAGCCACTCTTTATGTAAGAACACATGGACATTATCCAATTCAT 2936

|.||||||||..|..|.||||||||||||||||||...||||||||.|||

SFVcae_LK3 2888 CTGTTGTAGCTGCCTTATATGTAAGAACACATGGATCATATCCAATACAT 2937

SFVcae_FV2014 2937 GAAATGGCTAATGTGTTGAGGGCCATAGTTACTCAAGAAGGTGTTGCTAC 2986

|||.||||||||||..|..||||..|.|||||.||||||||.||.||.||

SFVcae_LK3 2938 GAATTGGCTAATGTTCTACGGGCAGTTGTTACCCAAGAAGGAGTAGCCAC 2987

SFVcae_FV2014 2987 TGGATTTACTATAGGCATGATGTTGTCAAACAATAATTATGATTTAATAT 3036

.||||||....|.||.||.|||||.||.||..|..|||||.||.|..|||

SFVcae_LK3 2988 AGGATTTCAGCTCGGGATTATGTTATCTAATCAAGATTATAATCTCGTAT 3037

SFVcae_FV2014 3037 GGGGAATTTTGCGACCCCTACTTCCTGGACAAGCTGTAGTCACTGCTATG 3086

||||||||||.||.|||||..|.|||||||||||||||||.||.||||||

SFVcae_LK3 3038 GGGGAATTTTACGTCCCCTTTTGCCTGGACAAGCTGTAGTTACAGCTATG 3087

SFVcae_FV2014 3087 CAACAAAGACTTGATAGAGAAGTTAATGATGCTGCTCGTATAGCATCTTT 3136

||.||||||||.|||..|||||||||||||||||||.|.||..|.||.||

SFVcae_LK3 3088 CAGCAAAGACTGGATCAAGAAGTTAATGATGCTGCTAGAATTACCTCCTT 3137

SFVcae_FV2014 3137 TGTTGGACATTTAAATGATATATATGGTCTCCTAGGTCTGAATGCTAGAG 3186

...|||||||||.||||||||||||...||||||||..|||||||..|||

SFVcae_LK3 3138 CAATGGACATTTGAATGATATATATCAACTCCTAGGGTTGAATGCCCGAG 3187

SFVcae_FV2014 3187 GTCAAAGTATTAATAGACCTCAGAGTGCATCAGCCTCAGGACCCTCTTCA 3236

|.|||||.|||..|||..|.||.|||||.||..||||.||....|||.|.

SFVcae_LK3 3188 GCCAAAGCATTGCTAGGGCCCAAAGTGCTTCGACCTCTGGGAATTCTGCC 3237

SFVcae_FV2014 3237 GCTACAGGTAGGGGAAGACAGAACCGTAGGAATCAACAACAGCCAGGACG 3286

.||.||||.||.|||||||.|...|..||.|.||||||.||..|.||.||

SFVcae_LK3 3238 TCTGCAGGAAGAGGAAGACGGGGGCAGAGAACTCAACAGCAAGCTGGCCG 3287

SFVcae_FV2014 3287 GCAGCAGCAACAACAGCCTCAAAGAAGAGGAAATCAAGGAACTCAAGGAC 3336

.||. ||||||||||....|||||||...||.||.||||.|||.||||

SFVcae_LK3 3288 TCAA---CAACAACAGCAAACAAGAAGAACTAACCAGGGAAATCAGGGAC 3334

SFVcae_FV2014 3337 AAAGGAATAATCAAAATAATCAGAGGCAGTCTTCAGGGGGTAATCAAGAT 3386

||||..|| ||||||||.||.||||||||.||.||||||||||..

SFVcae_LK3 3335 AAAGAGAT------AATAATCAAAGACAGTCTTCTGGAGGTAATCAAGGA 3378

SFVcae_FV2014 3387 CAAGGAGGACAGGGAGGATATAACCTAAGACCCAGAACTTATCAGCCTCA 3436

||..|||||||||||||||||.||.|||||||.|||||.||||||||.||

SFVcae_LK3 3379 CAGAGAGGACAGGGAGGATATGACTTAAGACCTAGAACGTATCAGCCACA 3428

SFVcae_FV2014 3437 GCGCTTTGGAGGAGGACGTGGTAGAAGATGGAATGATAATCAGCA---GC 3483

..|.|..|||||||||||.||.|||||||||||.|||||.||||| ||

SFVcae_LK3 3429 AAGATACGGAGGAGGACGAGGAAGAAGATGGAACGATAACCAGCAACAGC 3478

SFVcae_FV2014 3484 AACAAGTACAACCGAGCAGATCAGCTGATCCGTCTCGTTCCCAGAGTCAG 3533

||||||.|||.||..||||||||.||||||...|||||||||||||||||

SFVcae_LK3 3479 AACAAGCACAGCCAGGCAGATCATCTGATCAACCTCGTTCCCAGAGTCAG 3528

SFVcae_FV2014 3534 CAACCGCAATCAGAGGCTCGTGGCGATCAGTCACGAACATCTGGTGCAGG 3583

|||||||||.|||||||||||||||||||||||||||||||||||||.||

SFVcae_LK3 3529 CAACCGCAACCAGAGGCTCGTGGCGATCAGTCACGAACATCTGGTGCTGG 3578

SFVcae_FV2014 3584 GCGCGGACAACAGGGAAGAGGGAACCAAAACCGAAACCCACGCCGGGTTG 3633

||||||.|||||.|||||||||||.|||||||||||.|.||||||||.||

SFVcae_LK3 3579 GCGCGGCCAACAAGGAAGAGGGAATCAAAACCGAAATCAACGCCGGGCTG 3628

SFVcae_FV2014 3634 ATGTTAACGCTGCTCGGAATGTGGATACTGTGACAGTGACCACAACTTCC 3683

|||..|||..|.||||||||||||||||||||||||..||||||||||||

SFVcae_LK3 3629 ATGCAAACAATACTCGGAATGTGGATACTGTGACAGCAACCACAACTTCC 3678

**| *pol***

SFVcae_FV2014 3684 TCCTCAAC---TAGTTCTAGTCAAA**ATG**GATCCTCTTCAACTCCTTCAGC 3730

|||||.|| ||||||..|||||||||||||||||.||||||||.||||

SFVcae_LK3 3679 TCCTCCACGGCTAGTTCAGGTCAAA**ATG**GATCCTCTACAACTCCTCCAGC 3728

***gag* |**

SFVcae_FV2014 3731 CCCTGGAAGCAGAAATCAAGGGGAC**TAA**ATTAAAAGCCCACTGGGATAGT 3780

|.||||||||||||||||||||||||||..|.|||||.||.|||||||||

SFVcae_LK3 3729 CTCTGGAAGCAGAAATCAAGGGGAC**TAA**GCTGAAAGCTCATTGGGATAGT 3778

SFVcae_FV2014 3781 GGTGCTACAGTTACCTGTGTTCCCCAAGCATTCTTGGAAGATGAGACACC 3830

||.||.|||.|.||.|||||.||.|||||.||.||.|||||.||...|||

SFVcae_LK3 3779 GGAGCCACAATAACTTGTGTCCCACAAGCCTTTTTAGAAGAAGAAGTACC 3828

SFVcae_FV2014 3831 CATTAAAAATATTTGGATAAAGACTATACATGGAGAAAAAGAGCAACCTG 3880

.||||||||.||||||||.||.||.||.|||||.||||||||.|||||||

SFVcae_LK3 3829 AATTAAAAACATTTGGATCAAAACCATTCATGGTGAAAAAGAACAACCTG 3878

SFVcae_FV2014 3881 TTTATTATTTGACTTTTAAAGTACAAGGAAGAAAGGTAGAAGCAGAAGTA 3930

||||.|||||.|||||||||.|||||||.|||||.||.||||||||||||

SFVcae_LK3 3879 TTTACTATTTAACTTTTAAAATACAAGGCAGAAAAGTGGAAGCAGAAGTA 3928

SFVcae_FV2014 3931 ATTTCTTCTCCTTATGACTATATTTTAGTCAGCCCATCCGATATACCTTG 3980

||||||||.||||||||||||||.||||||||||||||||||||.|||||

SFVcae_LK3 3929 ATTTCTTCCCCTTATGACTATATATTAGTCAGCCCATCCGATATCCCTTG 3978

SFVcae_FV2014 3981 GCTAATGAAAAAGCCCCTTCAGTTGACTACTCTAGTACCACTCCAGGAAT 4030

|.|||||||.||.||.|||||.|||||.||||||||.||.||.|||||||

SFVcae_LK3 3979 GTTAATGAAGAAACCTCTTCAATTGACAACTCTAGTTCCTCTTCAGGAAT 4028

SFVcae_FV2014 4031 ATCAAGAAAGACTTTTAAAACAAACTGCATTACCAGAAAGAGAAAAGAAG 4080

||.|||||||||||||||||||.|||...|||.|.|.|||..|.||..|.

SFVcae_LK3 4029 ATGAAGAAAGACTTTTAAAACAGACTATGTTAACCGGAAGCTATAAAGAA 4078

SFVcae_FV2014 4081 ATATTACATTCTTTATTTTTGAAGTATGATGCATTATGGCAACATTGGGA 4130

|.||||||.|||||||||||||||||||||||||||||||||||||||||

SFVcae_LK3 4079 AAATTACAGTCTTTATTTTTGAAGTATGATGCATTATGGCAACATTGGGA 4128

SFVcae_FV2014 4131 GAACCAAGTAGGTCATAGGCGTATTAAGCCTCATCATATTGCAACTGGTA 4180

.||.||||||||.||||||||.|||||||||||.|||||.||||||||||

SFVcae_LK3 4129 AAATCAAGTAGGCCATAGGCGAATTAAGCCTCACCATATAGCAACTGGTA 4178

SFVcae_FV2014 4181 CAGTTAATCCAAGGCCACAAAAACAATATCCAATTAATCCTAAAGCAAAG 4230

||||||||||.||.||||||||||||||||||||.||.||.||||||||.

SFVcae_LK3 4179 CAGTTAATCCCAGACCACAAAAACAATATCCAATCAACCCAAAAGCAAAA 4228

SFVcae_FV2014 4231 CCTAGTATTCAAATTGTTATTAATGATTTACTAAAACAAGGTGTATTAAT 4280

.|.||.|||||||..|||||||||||||||||||||||||||||.|||||

SFVcae_LK3 4229 GCAAGCATTCAAACAGTTATTAATGATTTACTAAAACAAGGTGTTTTAAT 4278

SFVcae_FV2014 4281 ACAGCAAAACAGTGTTATGAATACTCCTGTGTATCCTGTCCCAAAGCCTG 4330

.||.|||||.|||.|.||||||||||||||.||.||.||.||.||.||.|

SFVcae_LK3 4279 TCAACAAAATAGTATAATGAATACTCCTGTATACCCAGTGCCTAAACCAG 4328

SFVcae_FV2014 4331 ATGGTAAATGGAGAATGGTATTAGATTATAGAGAAGTAAATAAAACTATT 4380

||||.||||||||||||||.|||||||||||||||||.||||||||.||.

SFVcae_LK3 4329 ATGGAAAATGGAGAATGGTTTTAGATTATAGAGAAGTTAATAAAACCATA 4378

SFVcae_FV2014 4381 CCTTTAATTGCTGCACAAAATCAACATTCGGCAGGAATATTGTCTTCTAT 4430

||||||||||||||.||.|||||.|||||.||.|||||.||.||.|||||

SFVcae_LK3 4379 CCTTTAATTGCTGCCCAGAATCAGCATTCTGCTGGAATTTTATCATCTAT 4428

SFVcae_FV2014 4431 TGTAAGAGAAAAATATAAAACAACTCTAGATTTATCTAATGGTTTTTGGG 4480

|.|.||||..|||||||||||.|||.|||||||.||||||||.||.||||

SFVcae_LK3 4429 TTTTAGAGGCAAATATAAAACCACTTTAGATTTGTCTAATGGCTTCTGGG 4478

SFVcae_FV2014 4481 CTCATTCCATTACTCCAGAATCATATTGGTTAACAGCCTTTACTTGGCAA 4530

||||.||.|||||.||||||||.|||||||||||.||.||||||||||..

SFVcae_LK3 4479 CTCACTCTATTACACCAGAATCTTATTGGTTAACTGCTTTTACTTGGCTT 4528

SFVcae_FV2014 4531 GGAAAACAATATTGCTGGACAAGACTCCCACAAGGATTTCTTAATAGCCC 4580

|||.||||||||||.|||||||||.|.||.||.||||||||||||||.||

SFVcae_LK3 4529 GGACAACAATATTGTTGGACAAGATTACCTCAGGGATTTCTTAATAGTCC 4578

SFVcae_FV2014 4581 TGCACTTTTTACAGCAGATGTTGTTGATTTGCTAAAAGAAGTACCAAATG 4630

|||..|.||||||||||||||.||||||||.||.||||||||||||||||

SFVcae_LK3 4579 TGCCTTATTTACAGCAGATGTAGTTGATTTACTTAAAGAAGTACCAAATG 4628

SFVcae_FV2014 4631 TACAAGCATATGTAGATGATATTTATATTAGTCATAATGACCCTAAAGAA 4680

|.||||..|||||.|||||.|||||||||||||||.|||||||||.||||

SFVcae_LK3 4629 TGCAAGTTTATGTGGATGACATTTATATTAGTCATGATGACCCTAGAGAA 4678

SFVcae_FV2014 4681 CATTTAGAACAACTTGAGAAAGTTTTTTCATTATTACTCAATGCTGGTTA 4730

||||||||||||||||||||||||||.||||||||.||||||||||||||

SFVcae_LK3 4679 CATTTAGAACAACTTGAGAAAGTTTTCTCATTATTGCTCAATGCTGGTTA 4728

SFVcae_FV2014 4731 TGTGGTTTCCTTAAAGAAGTCTGAAATTGCTCAATATGAAGTTGAATTTT 4780

|||||||||..|.||.||.|||||||||||||||.||||.|||||||||.

SFVcae_LK3 4729 TGTGGTTTCTCTTAAAAAATCTGAAATTGCTCAACATGAGGTTGAATTTC 4778

SFVcae_FV2014 4781 TGGGGTTCAATATTACAAAAGAAGGCCGAGGCCTAACTGATACTTTTAAG 4830

|.|||||.||||||||.|||||||||||||||||.|||||.|||||.||.

SFVcae_LK3 4779 TTGGGTTTAATATTACCAAAGAAGGCCGAGGCCTGACTGAAACTTTCAAA 4828

SFVcae_FV2014 4831 CAGAAATTGCTAAATATCACTCCACCTAAAGATCTTAAACAATTACAAAG 4880

||.|||.|..|||||||.||||||||.|.||||.|.||||||||.|||||

SFVcae_LK3 4829 CAAAAACTTTTAAATATTACTCCACCAAGAGATTTAAAACAATTGCAAAG 4878

SFVcae_FV2014 4881 TATTTTAGGATTGTTAAATTTTGCTAGAAATTTTATTCCTAATTTTTCTG 4930

|||||||||..|..||||||||||.||||||||||||||.||||||||||

SFVcae_LK3 4879 TATTTTAGGCCTTCTAAATTTTGCAAGAAATTTTATTCCCAATTTTTCTG 4928

SFVcae_FV2014 4931 AATTAGTTAAGCCATTATATAATATAATTGCTATTGCAAATGGTAAATTT 4980

||||||||||.||.||.||.|||||||||||||.||||||||||||||.|

SFVcae_LK3 4929 AATTAGTTAAACCTTTGTACAATATAATTGCTACTGCAAATGGTAAATAT 4978

SFVcae_FV2014 4981 ATTCAATGGACTGAAGAGAATAGTCAACAATTACAATACATTATTTCTGT 5030

|||..|||||||..|||.||||||||||||||||||.||||.|||||..|

SFVcae_LK3 4979 ATTACATGGACTACAGATAATAGTCAACAATTACAAAACATAATTTCCAT 5028

SFVcae_FV2014 5031 GTTAAATTCAGCTGAAAATTTAGAAGAAAGAAACCCTGAAGTAAAATTGA 5080

|||||||||.||.||||||.|.||||||||.||.||.|||||...|||.|

SFVcae_LK3 5029 GTTAAATTCTGCAGAAAATCTGGAAGAAAGGAATCCAGAAGTTCGATTAA 5078

SFVcae_FV2014 5081 TTATGAAAGTCAATACCTCTCCATCTGCTGGATATATTAGATTCTATAAT 5130

|.||||||||.||||||||.||.|||||.||||||||.|||||.||||||

SFVcae_LK3 5079 TAATGAAAGTAAATACCTCCCCTTCTGCAGGATATATAAGATTTTATAAT 5128

SFVcae_FV2014 5131 GAGTCAGCCAAAAGGCCAATAATGTATTTAAATTATGTGTACACAAAAGC 5180

||.|..||.||.||.|||||.|||||||||||||||||.||.||||||||

SFVcae_LK3 5129 GAATTTGCTAAGAGACCAATTATGTATTTAAATTATGTATATACAAAAGC 5178

SFVcae_FV2014 5181 TGAAATTAAATTCACTAATACTGAAAAGCTTTTAACTACCATACATAAAG 5230

.|||.|.|||||.|||||||||||.||.||.|||||.||.||||||||||

SFVcae_LK3 5179 AGAAGTCAAATTTACTAATACTGAGAAACTGTTAACCACAATACATAAAG 5228

SFVcae_FV2014 5231 GTCTTATTAAAGCTCTAGACCTAGCAATGGGTCAAGGTATTCTAGTATAT 5280

||||.|||||.|||.|||||||.|..||||||||||..|||.||||.||.

SFVcae_LK3 5229 GTCTAATTAAGGCTTTAGACCTTGGCATGGGTCAAGAAATTTTAGTCTAC 5278

SFVcae_FV2014 5281 AGTCCTATAGTCTCTATGACTAAAATTCAAAGAACGCCATTACCTGAAAG 5330

||.|||||.||.||.|||||||||||.||||.|||.||.|||||.||.||

SFVcae_LK3 5279 AGCCCTATTGTGTCCATGACTAAAATCCAAAAAACACCTTTACCAGAGAG 5328

SFVcae_FV2014 5331 AAAAGCTTTGCCAATTAGATGGATCACTTGGATGTCATACTTAGAGGATC 5380

||||||..|.||.||.||||||||.|||||||||||.||..|||||||||

SFVcae_LK3 5329 AAAAGCACTACCCATCAGATGGATAACTTGGATGTCTTATCTAGAGGATC 5378

SFVcae_FV2014 5381 CTAGAATTCAATTTCATTATGATAAAACATTACCTGAATTACAAAATGTG 5430

|.|||||.|||||.||||||||||||||.|||||||||.|||||.|.||.

SFVcae_LK3 5379 CCAGAATACAATTCCATTATGATAAAACTTTACCTGAACTACAACAAGTT 5428

SFVcae_FV2014 5431 CCTATGGTAACTGGAGATGAAGTGGCAAAAACAAAACATCCCAGTGAGTT 5480

||||..||.||.|..|||..|.|.||.||||..||||||||.|||||.||

SFVcae_LK3 5429 CCTACTGTTACAGATGATATAATAGCTAAAATTAAACATCCTAGTGAATT 5478

SFVcae_FV2014 5481 TTCCATGGTTTTTTATACTGATGGTTCAGCTATTAAGCATCCCAATATTA 5530

|...|||||||||||||||||.||.||.||.|||||.|||||.|||.|||

SFVcae_LK3 5479 TAGTATGGTTTTTTATACTGACGGATCTGCAATTAAACATCCTAATGTTA 5528

SFVcae_FV2014 5531 ATAAATCTCATAGTGCTGGAATGGGCATTGCCCAAGTCCAGTTTAAACCT 5580

||||.||.||||.||||||||||||.|||||.||.||.||||||||||||

SFVcae_LK3 5529 ATAAGTCACATAATGCTGGAATGGGTATTGCTCAGGTACAGTTTAAACCT 5578

SFVcae_FV2014 5581 GAATTTACTGTTTTAAATACTTGGTCTATTCCTTTGGGAGATCATACAGC 5630

||.|||||.||..|.|||||||||||.||||||.|.|||||||||||.||

SFVcae_LK3 5579 GAGTTTACAGTAATTAATACTTGGTCAATTCCTCTTGGAGATCATACTGC 5628

SFVcae_FV2014 5631 TCAGCTGGCAGAAGTAGCAGCTGTGGAGTTTGCATGCAAAAAGGCCCTTA 5680

.||.||.||||||||.|||||||||||.||||||||||||||||||||||

SFVcae_LK3 5629 ACAACTTGCAGAAGTTGCAGCTGTGGAATTTGCATGCAAAAAGGCCCTTA 5678

SFVcae_FV2014 5681 AAATAAATGGGCCTGTATTAATAGTCACTGACAGTTTCTATGTTGCTGAG 5730

|||||.||||.||||||||||||||.|||||.|||||.|||||||||||.

SFVcae_LK3 5679 AAATAGATGGACCTGTATTAATAGTTACTGATAGTTTTTATGTTGCTGAA 5728

SFVcae_FV2014 5731 AGTGCTAATAAAGAATTGCCATATTGGCAATCTAATGGGTTCCTTAACAA 5780

||||.||||||.||||||||||||||||||||.||||||||..||||.||

SFVcae_LK3 5729 AGTGTTAATAAGGAATTGCCATATTGGCAATCAAATGGGTTTTTTAATAA 5778

SFVcae_FV2014 5781 TAAAAAGAAACCTCTTAGACATATTTCTAAATGGAAATCAATTGCTGAAT 5830

||||||||||||..|.|.||||.|.|||||.||||||||||||||.||.|

SFVcae_LK3 5779 TAAAAAGAAACCCTTAAAACATGTCTCTAAGTGGAAATCAATTGCAGATT 5828

SFVcae_FV2014 5831 GCATACAGTTAAAGCCTGATATTTCTATTATACATGAAAAAGGACACCAG 5880

|.|||||||||||.||.||||||..||||||.|||||||||||.||||||

SFVcae_LK3 5829 GTATACAGTTAAAACCAGATATTATTATTATTCATGAAAAAGGTCACCAG 5878

SFVcae_FV2014 5881 CCCACTGCCACCACCTTCCATACAGAAGGAAATACTTTGGCTGATAAGCT 5930

||.|||||..|.|||||.|||||||||||.||||.|||.||||||||.||

SFVcae_LK3 5879 CCTACTGCTTCTACCTTTCATACAGAAGGTAATAATTTAGCTGATAAACT 5928

SFVcae_FV2014 5931 TGCCACCCAAGGGAGTTATGTGGTAAATTCAAACACCACGCCAAGCCTGG 5980

||||||||||||||||||||||||||||..|||.|||||.||||||||||

SFVcae_LK3 5929 TGCCACCCAAGGGAGTTATGTGGTAAATATAAATACCACTCCAAGCCTGG 5978

SFVcae_FV2014 5981 ATGCAGAGTTGGATCAATTATTACAAGGACGATATCCTAAAGGTTTTCCA 6030

||||||||||||||||||||.|||||||||.||||||||||||.||||||

SFVcae_LK3 5979 ATGCAGAGTTGGATCAATTACTACAAGGACAATATCCTAAAGGCTTTCCA 6028

SFVcae_FV2014 6031 AAACAATATATATATAAATTACAAGGTGGACAAGTTATTGTCTCTCGTCC 6080

|||||.|||..||||.||.|..||..||||||||||||.||..|||||||

SFVcae_LK3 6029 AAACATTATCAATATCAACTTGAAAATGGACAAGTTATGGTAACTCGTCC 6078

SFVcae_FV2014 6081 ACAAGGAGATCGTATTATTCCTCCTAAATCTGACAGGCCTCAACTTATTT 6130

|.|.|||.|..|.|||||||||||.|||||.||||||||||||.|.||||

SFVcae_LK3 6079 AAATGGAAAGAGAATTATTCCTCCAAAATCAGACAGGCCTCAAATCATTT 6128

SFVcae_FV2014 6131 TACAAGCTCATAATATTGCTCATACAGGAAGAGATTCAACCTTTCTTAAG 6180

|||||||.|||||||||||.||.||||||||||||||||||||||||||.

SFVcae_LK3 6129 TACAAGCACATAATATTGCACACACAGGAAGAGATTCAACCTTTCTTAAA 6178

SFVcae_FV2014 6181 GTCTCTTCCAAGTATTGGTGGCCAAATCTTAGAAAGGATGTGGTTAAAGT 6230

||||||||||||||||||||||||||||||||||||||||||||||||||

SFVcae_LK3 6179 GTCTCTTCCAAGTATTGGTGGCCAAATCTTAGAAAGGATGTGGTTAAAGT 6228

SFVcae_FV2014 6231 TATTAGACAGTGTAAACAATGTCTGGTCACCAATGCAGCTGTTCTAGCTG 6280

|||.|||||.|||||.||||||||||||||.|||||||||..||||||||

SFVcae_LK3 6229 TATCAGACAATGTAAGCAATGTCTGGTCACAAATGCAGCTACTCTAGCTG 6278

SFVcae_FV2014 6281 CACCTCCAATACTGAGGCCTGACAGACCAATTAAGCCTTTTGATAAATTT 6330

|.|||||.|||.||||||||||..||||..|.||||||||||||||||||

SFVcae_LK3 6279 CGCCTCCGATATTGAGGCCTGAACGACCTGTAAAGCCTTTTGATAAATTT 6328

SFVcae_FV2014 6331 TTCATTGATTATATTGGACCTTTACCCCCTTCTAATGGATATCTTCATGT 6380

||.||||||||||||||.||.||||||||.||||||||||||.|.|||||

SFVcae_LK3 6329 TTTATTGATTATATTGGTCCATTACCCCCCTCTAATGGATATTTACATGT 6378

SFVcae_FV2014 6381 CCTTGTAGTAGTCGATGGTATGACTGGTTTTGTTTGGTTATACCCCACAA 6430

||||||||||||||||.||||||||||.||||||||||||||||||||.|

SFVcae_LK3 6379 CCTTGTAGTAGTCGATAGTATGACTGGATTTGTTTGGTTATACCCCACTA 6428

SFVcae_FV2014 6431 AGGCTCCTTCAACTAGCGCAACTGTTAAAGCTCTCAATATGCTCACTAGT 6480

|||||||||||||||||||||||||||||||||||||.||||||||||||

SFVcae_LK3 6429 AGGCTCCTTCAACTAGCGCAACTGTTAAAGCTCTCAACATGCTCACTAGT 6478

SFVcae_FV2014 6481 ATTGCAGTTCCAAAGGTGATACACTCTGATCAGGGTGCTGCATTCACCTC 6530

||||||||||||||||||||||||||||||||||||||.|||||||||||

SFVcae_LK3 6479 ATTGCAGTTCCAAAGGTGATACACTCTGATCAGGGTGCAGCATTCACCTC 6528

SFVcae_FV2014 6531 TGCAACCTTTGCTGAATGGGCAAAGGACAAAGGTATACACTTGGAATTCA 6580

||||||.||||||||.||||||||..|||||||||||||.||||||||||

SFVcae_LK3 6529 TGCAACTTTTGCTGATTGGGCAAAAAACAAAGGTATACAATTGGAATTCA 6578

SFVcae_FV2014 6581 GTACTCCTTACCACCCTCAAAGTAGTGGGAAGGTGGAAAGGAAAAATAGT 6630

||||||||||||||||.|||||||||||.|||||||||||||||||||||

SFVcae_LK3 6579 GTACTCCTTACCACCCCCAAAGTAGTGGCAAGGTGGAAAGGAAAAATAGT 6628

SFVcae_FV2014 6631 GACATAAAACGACTATTAACTAAACTGTTAGTTGGGAGACCTGCTAAGTG 6680

||||||||||||||.||||||||||||.|.||||||||||||||||||||

SFVcae_LK3 6629 GACATAAAACGACTTTTAACTAAACTGCTTGTTGGGAGACCTGCTAAGTG 6678

SFVcae_FV2014 6681 GTATGATCTTCTACCAGTTGTACAATTGGCATTAAATAATTCTTATAGTC 6730

||||||||||||.|||||||||||||||||||||||||||||||||||||

SFVcae_LK3 6679 GTATGATCTTCTTCCAGTTGTACAATTGGCATTAAATAATTCTTATAGTC 6728

SFVcae_FV2014 6731 CAGCTTCTAAATATACTCCTCATCAACTTCTGTTTGGTATAGATTCAAAT 6780

|..||||||||||||||||||||||||||.|||||||||||||||||||.

SFVcae_LK3 6729 CTTCTTCTAAATATACTCCTCATCAACTTTTGTTTGGTATAGATTCAAAC 6778

SFVcae_FV2014 6781 ACTCCATTTGCAAATTCAGATACACTTGACTTATCAAGAGAAGAAGAACT 6830

||.||||||||||||||.|||||||||||.||||||||||||||||||||

SFVcae_LK3 6779 ACACCATTTGCAAATTCTGATACACTTGATTTATCAAGAGAAGAAGAACT 6828

SFVcae_FV2014 6831 CTCTCTTTTACAGGAAATCAGAACTTCTCTTCACCATCCTACCTCCCCAC 6880

||||||||||||||||||||||.|.||||||.|||.|||..||.||||.|

SFVcae_LK3 6829 CTCTCTTTTACAGGAAATCAGATCCTCTCTTTACCTTCCGTCCACCCCTC 6878

SFVcae_FV2014 6881 CATCCTCCGTCCGTGCTTGGTCTCCTTCTGTTGGCCAGTTGGTCCAGGAG 6930

|..|||||.|.|||||.||||||||||||||||||||.||.|||||||||

SFVcae_LK3 6879 CTGCCTCCATTCGTGCCTGGTCTCCTTCTGTTGGCCAATTAGTCCAGGAG 6928

SFVcae_FV2014 6931 AGGGTGGCTAGGCCCGCATCCTTGCGACCACGGTGGCATAAACCTACTAA 6980

|||||.||.|||||.||||||||..||||.||||||||||||||.|||..

SFVcae_LK3 6929 AGGGTAGCCAGGCCTGCATCCTTAAGACCTCGGTGGCATAAACCAACTCC 6978

SFVcae_FV2014 6981 GGTCTTGGAAGTTCTGAATCCCCGCACTGTTGTTATTTTGGACCATCTTG 7030

.||.|||||||||.|.|||||.||..||||||||||||||||||||||||

SFVcae_LK3 6979 TGTTTTGGAAGTTATTAATCCACGGGCTGTTGTTATTTTGGACCATCTTG 7028

SFVcae_FV2014 7031 GCAACAGGAGAACTGTAAGTGTTGATAATTTGAAGTTAACTGTTTATCAG 7080

|||||||.||||||||||||||.||.|||.|||||.||||||.|||||||

SFVcae_LK3 7029 GCAACAGAAGAACTGTAAGTGTGGACAATCTGAAGCTAACTGCTTATCAG 7078

**|*env***

SFVcae_FV2014 7081 AACA**ATG**GCTCCTCCAATGAATCTTCAGCAGTGGCTGCTATGGAAGAAAA 7130

||..|||||.||.|||||||||||.|||||||||.||||||||||.||.|

SFVcae_LK3 7079 AAGG**ATG**GCACCCCCAATGAATCTGCAGCAGTGGTTGCTATGGAAAAAGA 7128

***pol*|**

SFVcae_FV2014 7131 TGAA**TGA**GGCACATATGGCCTTGGAACATGTTTCAACTCTTACTGAAGAA 7180

|||||||..|.||..||||.||||||.||.|||||.|.||||||||||||

SFVcae_LK3 7129 TGAA**TGA**AACCCACTTGGCGTTGGAAAATATTTCATCCCTTACTGAAGAA 7178

SFVcae_FV2014 7181 CAGAAACAGCAGGTAATTGTGGAAATCCAGCAGGAAGAAGTAATACCAAC 7230

||.||.||.||.||.|||.|.|||||.||.||.||||||||.|||||.||

SFVcae_LK3 7179 CAAAAGCAACAAGTGATTATTGAAATTCAACAAGAAGAAGTGATACCTAC 7228

SFVcae_FV2014 7231 CAAAATGGACAGACTAAAATATCTAGCATACGCCTGTTGTGCTACTAGTA 7280

.|..|||||||||.||||.|||||||||||.||.||||||||||||||||

SFVcae_LK3 7229 TAGGATGGACAGAGTAAAGTATCTAGCATATGCATGTTGTGCTACTAGTA 7278

SFVcae_FV2014 7281 CACGTGTTATGTGTTGGTTGTTATTAGTCTGTGTCTTATTAATTATTGTA 7330

|.|||||.|||||||||.|.||.||..|.|||||.|||||||||||||||

SFVcae_LK3 7279 CCCGTGTCATGTGTTGGCTATTTTTGATTTGTGTATTATTAATTATTGTA 7328

SFVcae_FV2014 7331 TTCATATCCTGTTTCGTTACTATTGCTAGATTACAATGGAATAGGGACAT 7380

||..||||.|||||.||.|||.|||||||..|.||||||||||||||.||

SFVcae_LK3 7329 TTTGTATCATGTTTTGTCACTGTTGCTAGGATTCAATGGAATAGGGATAT 7378

SFVcae_FV2014 7381 TGTTACTTTAGGACCTGTCATAGACTGGAATGTAACTCATCAAGCTACAT 7430

|..|...||.||.||.|||||.||.||||||||.||||||||.||.||||

SFVcae_LK3 7379 TAATGTATTTGGTCCAGTCATTGATTGGAATGTTACTCATCAGGCCACAT 7428

SFVcae_FV2014 7431 ATCAACAGCTTAGAGCGGCCAGGTTGACAAGGTCATTGAGGGTTGAACAT 7480

|.|||||.||||.|||.||.||..|.||.||.||.||||.||||||.|||

SFVcae_LK3 7429 ACCAACAACTTAAAGCTGCTAGACTAACTAGATCCTTGAAGGTTGAGCAT 7478

SFVcae_FV2014 7481 CCATATATTTCATATATATCTTTGAATATTTCAAGTATACCACAAGGTAT 7530

||..||||.||.|||||||||.|.|||||.||.||.||||||||||||.|

SFVcae_LK3 7479 CCTCATATATCGTATATATCTATAAATATGTCCAGCATACCACAAGGTGT 7528

SFVcae_FV2014 7531 TATATATGTTCCTCATCCAGAACCCATAATCCTCAAGGAGAGGGTTTTAG 7580

|||.|||...|||||.||.|||||.|||||.|||||||||||||||.|||

SFVcae_LK3 7529 TATGTATACACCTCACCCTGAACCTATAATTCTCAAGGAGAGGGTTCTAG 7578

SFVcae_FV2014 7581 GATTGTCACAGGTCCTATTGATAAATTCGGAAAATATAGCCAATGTAGCT 7630

||.|.|||||.||..||.||||||||||.|||||||||||.|||||||||

SFVcae_LK3 7579 GAATATCACAAGTGTTAATGATAAATTCTGAAAATATAGCTAATGTAGCT 7628

SFVcae_FV2014 7631 AATTTGACACAAGAAACAAAGATATTATTAACAGATATGATTAATGAAGA 7680

||.|||.||||||||||.|||.|||||.||||.||||||||.||||||||

SFVcae_LK3 7629 AACTTGTCACAAGAAACTAAGGTATTACTAACTGATATGATAAATGAAGA 7678

SFVcae_FV2014 7681 ATTGCAAGATTTATCTAACCAAATGATTGATTTTGAACTACCTTTGGGAG 7730

|||.||||||||||||||.|||||||||||.||||||.|||||.|.||.|

SFVcae_LK3 7679 ATTACAAGATTTATCTAATCAAATGATTGACTTTGAATTACCTCTAGGGG 7728

SFVcae_FV2014 7731 ACCCCCGTGATCAAGAGCAATACATACATCATAAATGTTATCAGGAGTTT 7780

|.||..|.||||||||.|||||.|||||||||||||||||.|||||.|||

SFVcae_LK3 7729 ATCCTAGAGATCAAGATCAATATATACATCATAAATGTTACCAGGAATTT 7778

SFVcae_FV2014 7781 GCTCATTGTTATTTAGTGAAATATAAAAATCCTAAAGCATGGAGTTCATC 7830

||||||||||||||.||.||.|||||.||.|||....|.||||..||...

SFVcae_LK3 7779 GCTCATTGTTATTTGGTTAAGTATAAGAAGCCTTCTCCTTGGATATCTGA 7828

SFVcae_FV2014 7831 CTCTCTAGTAGCAGATCAATGTCCATTACCTGGAAACCATCCAACTGTCC 7880

...|.|..|.|.|||||||||.||.||.|||.|||..|||....||..|.

SFVcae_LK3 7829 GGGTATTATTGTAGATCAATGCCCTTTGCCTAGAATACATGATCCTAACT 7878

SFVcae_FV2014 7881 AATATGCCTATCAACAAATATGGGACTATTATGTCCCGTTTCAACAAATA 7930

|.|||...|||||||..||.||||||||||||.|.....|||||.|.||.

SFVcae_LK3 7879 ATTATAAATATCAACCTATCTGGGACTATTATTTGAAAATTCAAAATATT 7928

SFVcae_FV2014 7931 AGACCAGAAGGATGGACCTCCGCTTCTGTTTATGAGGACGCCAGAATAGG 7980

||.||..|.||.|||||.||....|||...||.|.....||.|||||.||

SFVcae_LK3 7929 AGGCCTCAGGGGTGGACTTCTAAATCTTACTACGGAACTGCTAGAATGGG 7978

SFVcae_FV2014 7981 AGGTTTTTATATCCCCAAGAGATATAGAAATTCTTCATATACCCATGTAT 8030

|.||||||||||.||.|.....|..||||||..|.||..||..|||||..

SFVcae_LK3 7979 AAGTTTTTATATTCCTACATTTTTGAGAAATAATACAGTTAGTCATGTGC 8028

SFVcae_FV2014 8031 TGTTTTGTTCAGACCAAATCTATAACAAATGGTATAATGTATTAAATACT 8080

|.||.|||||.||.||..|.|||...||||||||||||.||..|||||.|

SFVcae_LK3 8029 TATTCTGTTCTGATCAGCTTTATGGTAAATGGTATAATATAGAAAATAAT 8078

SFVcae_FV2014 8081 GTAAAAGAAAATGAGGACTTATTGGTAACAAAATTATATAACATAACTAA 8130

.||.|.||||||||..|.||.|||..||||||.||||||||..|.||||.

SFVcae_LK3 8079 ATACAGGAAAATGAACAATTGTTGAAAACAAAGTTATATAATTTGACTAC 8128

SFVcae_FV2014 8131 AGTAGGAACTGCTCAACTTAAAGATAGAGGATTACCTCCAAGTTGGAATA 8180

| || |..|.||.|.||.|.|||||..|||||...|...|||||||

SFVcae_LK3 8129 A-TA-----TAGTAAATTGAAGGCTAGAGCTTTACCAAAAGAATGGAATA 8172

SFVcae_FV2014 8181 GGAATGGAAAAAGTTCCTTTTTTAGGGAAATTAACACATTGGATATATGT 8230

...|.||.||...|...||.||.|||....||||..|..|||||.|||||

SFVcae_LK3 8173 ATCAAGGGAATGCTAGATTATTCAGGAGTTTTAATCCTCTGGATGTATGT 8222

SFVcae_FV2014 8231 AATAGACCTGAAATGGTTCTATTATTAAATTCATCATATTTTTCCTTCTC 8280

|||.||||.|||...||..||.||||.|||.|..|.|||||..|.|..||

SFVcae_LK3 8223 AATCGACCAGAAGCAGTATTACTATTGAATACTACTTATTTCACTTATTC 8272

SFVcae_FV2014 8281 ATTATGGGAAGGAGACTGTGGATATACAAGAGAGAATGTCACACAAGCTC 8330

.|||||||||||.||.|||...|||||.|..| .|.|.|..|||..||

SFVcae_LK3 8273 CTTATGGGAAGGTGATTGTAATTATACTACTG---CTCTTATTCAAAATC 8319

SFVcae_FV2014 8331 ACCCTCTTTGTAAAGATTTTTATAATAATTCAAGAAAACATTGGCATCCA 8380

.|.|....||||.|.|.....|||. |||.|||..||.| ||.||.

SFVcae_LK3 8320 TCACAGAGTGTAGACAACCAGATAG--ATTAAAGCTAAAA----CACCCT 8363

SFVcae_FV2014 8381 TACTCTTGTAGATTCTGGAGGTATAAAAATGAAAAAGAAGAAGTTAAATG 8430

||..|.|||||.||.|||||.||||||.|.|...||||||||||.|||||

SFVcae_LK3 8364 TATGCATGTAGGTTTTGGAGATATAAAGAAGGCCAAGAAGAAGTAAAATG 8413

SFVcae_FV2014 8431 TAGGAATGAAGATAAAAAAAGATGCATATATTATCCATTATGGGATACTC 8480

|.....|.|.||.|||||.|..||..|.||||||.|...||.....|..|

SFVcae_LK3 8414 TTTAGGTAATGAGAAAAAGAAGTGTCTCTATTATTCTGAATATTCAAGCC 8463

SFVcae_FV2014 8481 CAGAAGCATTGTATGATTTTGGATTTCTTGCTTATCTAAATGCATTTCCT 8530

|.|||||...||.||||||||||||||||.|.|||.|||||||.||||||

SFVcae_LK3 8464 CGGAAGCCCAGTTTGATTTTGGATTTCTTTCATATTTAAATGCTTTTCCT 8513

SFVcae_FV2014 8531 TCTCCAAGTTGTGTTCAAAATAATACTATAAGAGAATCTGAATATGAAGT 8580

......|..|...||.|||||.|.||..|.||.|||.|.|||||||||||

SFVcae_LK3 8514 GGATTGAAATACATTGAAAATCAGACAGTTAGGGAACCAGAATATGAAGT 8563

SFVcae_FV2014 8581 TCATTCCATATATATGGAATGTATGAATGCAGCCAAAAAACATGATATAG 8630

..||||..||||||||||||||||||||.|.|||.||||..|||..||||

SFVcae_LK3 8564 ATATTCTTTATATATGGAATGTATGAATTCTGCCGAAAAGTATGGGATAG 8613

SFVcae_FV2014 8631 ATAGTGTATTATTTGCACTGAAAACCTTTTTGAATTTTACTGGAACTCCT 8680

|||||||.||||||||..|.||||||||.||.||||||||||||||.||.

SFVcae_LK3 8614 ATAGTGTTTTATTTGCTTTAAAAACCTTCTTAAATTTTACTGGAACACCA 8663

SFVcae_FV2014 8681 GTAAATGAAATGCCTACAGCTAGGGCTTTTGTAGGCCTTACAGATCCTAA 8730

||.|||||||||.|.||.|||||.||.||||||||||||||||||||.||

SFVcae_LK3 8664 GTGAATGAAATGTCAACTGCTAGAGCATTTGTAGGCCTTACAGATCCCAA 8713

SFVcae_FV2014 8731 ATTCCCTCCTGTTTATCCCAATGTGACAAGAGAACAAAAAGGTTGTGA-A 8779

|||.|||||....|||||.|||.|.||.|.||||||||||.|.||..| |

SFVcae_LK3 8714 ATTTCCTCCAACATATCCAAATATTACTAAAGAACAAAAAAGATGCAACA 8763

SFVcae_FV2014 8780 TCTTCAA--GAAAGAAAAGAAGCACTAACATAGAAAAGCTTAGATCAATG 8827

.|||.|| |||.|||||||||.|||||.||.|||||.||||||||||||

SFVcae_LK3 8764 ACTTAAAAAGAAGGAAAAGAAGTACTAATATTGAAAAACTTAGATCAATG 8813

SFVcae_FV2014 8828 GGATATGCACTTACTGGAGCAGTACAAACCTTGTCTCAAATTTCTGACAT 8877

||||||.||.|.||||||||.||||||||..|.||.|||||.||.||.||

SFVcae_LK3 8814 GGATATTCATTAACTGGAGCTGTACAAACTCTTTCACAAATATCAGATAT 8863

SFVcae_FV2014 8878 TAATGATGAAAAGTTACAACAAGGAGTTTATTTACTAAGAAACCATGTTG 8927

.||||||||||.|||||||||||||||.|..|||.|||||.|.|||||||

SFVcae_LK3 8864 AAATGATGAAAGGTTACAACAAGGAGTATCATTATTAAGAGATCATGTTG 8913

SFVcae_FV2014 8928 TCACTCTAATGGAAGCTGCATTACATGATATTACTATCATGGAAGGAATG 8977

|||||.|.|||||||||||..||||||||||.||.||.||||||||||||

SFVcae_LK3 8914 TCACTTTGATGGAAGCTGCCCTACATGATATCACCATTATGGAAGGAATG 8963

SFVcae_FV2014 8978 TTAGCCATTCAGCATGTGCATACTCATTTGACTCATCTTAAAACAATGTT 9027

.||||.||.|||||||||||.||||||||||.||||||||||||.||.||

SFVcae_LK3 8964 CTAGCAATCCAGCATGTGCACACTCATTTGAATCATCTTAAAACTATATT 9013

SFVcae_FV2014 9028 GCTGATGAGGAAGATAGATTGGACATTTATTAAAAGTAATTGGATTCAAG 9077

..|.|||||.|||||.|||||||||||||||||||||||.||||||.|||

SFVcae_LK3 9014 ATTAATGAGAAAGATTGATTGGACATTTATTAAAAGTAACTGGATTAAAG 9063

SFVcae_FV2014 9078 AACAGTTACAAAAATCTGAAGATGAAATGAAGATTATAAGAAGAACGGCC 9127

||||..|.|||||..|||||||||||||||||||.||.||||||||.||.

SFVcae_LK3 9064 AACAACTCCAAAAGACTGAAGATGAAATGAAGATCATTAGAAGAACAGCT 9113

SFVcae_FV2014 9128 AAAAGTCTAGTATATTATGTTACTCAAACATCATCATCACCTACAGCTAC 9177

||||||.|||||||.|||||.||||||||||||||.||..|||||||.||

SFVcae_LK3 9114 AAAAGTTTAGTATACTATGTAACTCAAACATCATCCTCTACTACAGCAAC 9163

SFVcae_FV2014 9178 TTCTTGGGAAATTGGAATCTATTATGAAATCACTATTCCTAAACATATTT 9227

.||.|||||.||||||||.||||||||.||.|||||.|||||||||||||

SFVcae_LK3 9164 ATCATGGGAGATTGGAATTTATTATGAGATAACTATACCTAAACATATTT 9213

SFVcae_FV2014 9228 ACCTTAACAATTGGCAAGTGATTAATATAGGTCATCTTGTTGAGTCAGCC 9277

|..|.||.||||||||.||.|||||||||||||||||||||||||||||.

SFVcae_LK3 9214 ATTTAAATAATTGGCAGGTTATTAATATAGGTCATCTTGTTGAGTCAGCT 9263

SFVcae_FV2014 9278 GGACACTTAACCTTAATTAAGGTCAAGCATCCTTTTGAGGTAATTAATAA 9327

||.||.|||||||||||.|.|||.||.|||||||.||||||.||||||||

SFVcae_LK3 9264 GGTCATTTAACCTTAATAAGGGTTAAACATCCTTATGAGGTCATTAATAA 9313

SFVcae_FV2014 9328 GGAATGCAAATATGAACAATATTTGCATCTTGAAGATTGTATATCCCAGG 9377

.|||||.|.|||||||||||||||.||.||||||||.||.|||||.||.|

SFVcae_LK3 9314 AGAATGTACATATGAACAATATTTACACCTTGAAGACTGCATATCTCAAG 9363

SFVcae_FV2014 9378 ATTATGTGATTTGTGATGTAGTACAAATAGTTTCACCATGTGGCAATTCA 9427

||||||||||||||||...||||||||||||.|||||||||||.|||||.

SFVcae_LK3 9364 ATTATGTGATTTGTGACACAGTACAAATAGTGTCACCATGTGGAAATTCG 9413

SFVcae_FV2014 9428 ACAATTACTAGTGACTGTCCAGTCACTGCTCAAAAGGTAAAAGAACCATA 9477

||||..||||||||||||||.|||||||||.|.||||||||.||||||||

SFVcae_LK3 9414 ACAACAACTAGTGACTGTCCTGTCACTGCTGAGAAGGTAAAGGAACCATA 9463

SFVcae_FV2014 9478 TATTCAGGTTTCTGCCTTAAAAAATGGTAGTTATCTGGTTTTAAGTAGTA 9527

|.||||.||.||.||.|||||||||||.||.|||.|.|||.|||.|||||

SFVcae_LK3 9464 TGTTCAAGTATCAGCTTTAAAAAATGGAAGCTATTTAGTTCTAACTAGTA 9513

SFVcae_FV2014 9528 GAAAAGATTGCTCAATACCAGCATATGTTCCCAGTGTAGTAACAGTTAAT 9577

|||.|||||||||||||||||||||||||||.|||.|.|||||.||.|||

SFVcae_LK3 9514 GAACAGATTGCTCAATACCAGCATATGTTCCTAGTATTGTAACTGTGAAT 9563

SFVcae_FV2014 9578 GAAACAGTCAGATGTTTTGGGGATGAGTTTCACAAACCGCTATATTCAGA 9627

||||||||.|..||||||||||.|||||||||.|||||.|||||||||||

SFVcae_LK3 9564 GAAACAGTTAAGTGTTTTGGGGTTGAGTTTCATAAACCACTATATTCAGA 9613

SFVcae_FV2014 9628 AACTAAAGTCAGCTTTGAACCACAAGTTCCACATCTGAAACTGCGCTTAC 9677

||.|||||||||||||||||||||||||||.|||||||||||.|||||.|

SFVcae_LK3 9614 AAGTAAAGTCAGCTTTGAACCACAAGTTCCGCATCTGAAACTACGCTTGC 9663

SFVcae_FV2014 9678 CACATCTAGTTGGAATAATTGCAAGTCTTCAGAATTTGGAAATTGAAGTA 9727

|||||||.|||||.||.|||||||.|||||||||||||||||||||||||

SFVcae_LK3 9664 CACATCTGGTTGGGATTATTGCAAATCTTCAGAATTTGGAAATTGAAGTA 9713

**TATA**

SFVcae_FV2014 9728 ACAAGCACTCAAGAAAGTATAAAAGATCAGATTGAAAGAGCAAAATCACA 9777

||.||||||||||||||||||||||||||||||||||||||.||||||||

SFVcae_LK3 9714 ACCAGCACTCAAGAAAGTATAAAAGATCAGATTGAAAGAGCTAAATCACA 9763

SFVcae_FV2014 9778 ACTTCTTCGACTGGACATCCACGAAGGAGACTTTCCTACTTGGATACAGC 9827

.||||||||.||||||||||||||||||||.||||||.|||||||.||.|

SFVcae_LK3 9764 GCTTCTTCGGCTGGACATCCACGAAGGAGATTTTCCTGCTTGGATTCAAC 9813

SFVcae_FV2014 9828 AACTAGCTTCAGCCACCAGGGACGTTTGGCCTGCTGCAGCTCAAGCTCTT 9877

||||.||.||.||.|||||.||||||||||||||.||||||..|||||||

SFVcae_LK3 9814 AACTTGCCTCTGCTACCAGAGACGTTTGGCCTGCAGCAGCTAGAGCTCTT 9863

SFVcae_FV2014 9878 CAAGGCATAGGTAACTTTTTGTCTAATACTGCCCAAGGGATATTTGGAAC 9927

|||||||||||||||.|||||||||||||||||||.||.|||||||||||

SFVcae_LK3 9864 CAAGGCATAGGTAACGTTTTGTCTAATACTGCCCAGGGAATATTTGGAAC 9913

SFVcae_FV2014 9928 AACTGTGAGTATCCTATCGTATGCAAAACCTATCCTAATAGGAATAGGTG 9977

.||.||.||.||..|.||.||||||||.||||||||.|||||||||||||

SFVcae_LK3 9914 TACAGTAAGCATTTTGTCTTATGCAAAGCCTATCCTGATAGGAATAGGTG 9963

**| *tas/bet***

SFVcae_FV2014 9978 TTATACTCTTGATTGCCTTTTTGTTTAAGATTGTATC**ATG**GCTTCCTGGG 10027

|||||||.||||||||||||||.|||||||||||||||||||||||||||

SFVcae_LK3 9964 TTATACTTTTGATTGCCTTTTTATTTAAGATTGTATC**ATG**GCTTCCTGGG 10013

***env* |**

SFVcae_FV2014 10028 AAAAAGAAAAAGAAT**TAA**CCCATCTCCATCAAGAGGGAGACGATCCACTG 10077

||||||||||.||||||.|.||||||||||||...|.|||.||.||..|.

SFVcae_LK3 10014 AAAAAGAAAAGGAAT**TAG**CTCATCTCCATCAACCAGAAGATGACCCTTTA 10063

SFVcae_FV2014 10078 CCAGATTTATCAATACTCCTGGATATGGATCAGTTAGAGCCCACTGAAGG 10127

||||||||.|||.|.||||||||||||||||||||.||||||||||||||

SFVcae_LK3 10064 CCAGATTTGTCATTGCTCCTGGATATGGATCAGTTTGAGCCCACTGAAGG 10113

SFVcae_FV2014 10128 GCCTGATTCAAATCCAGGAGCTGAAAAAATCTATCTACAACTACAAACAA 10177

||||||||||||||||||||||||.||.||.|||||||||.|||||..|.

SFVcae_LK3 10114 GCCTGATTCAAATCCAGGAGCTGAGAAGATTTATCTACAATTACAAGTAG 10163

SFVcae_FV2014 10178 TGCCTGGAGATTCATCTGAAAAAACTTACAGATTTGGCTATGAAGAAAAA 10227

..||.||||||.|.||.||||||||.||.|.||||||.||||||||.|||

SFVcae_LK3 10164 CTCCAGGAGATCCTTCAGAAAAAACATATAAATTTGGATATGAAGATAAA 10213

SFVcae_FV2014 10228 GAAGCTCAAAACCCAGATTTAAAAATGAGAAATTGGGTTCCGGATCCTGA 10277

|||||||||||.||.||||||||.|||||||||||||||||.|||||.||

SFVcae_LK3 10214 GAAGCTCAAAATCCTGATTTAAAGATGAGAAATTGGGTTCCTGATCCAGA 10263

SFVcae_FV2014 10278 TAAAATGAGTAAGTGGGCCTGTGCAAGGCTTATTCTTTGTGGACTTTATA 10327

.||||||||||||||||||||||||||||||||||||||||||||.||||

SFVcae_LK3 10264 AAAAATGAGTAAGTGGGCCTGTGCAAGGCTTATTCTTTGTGGACTATATA 10313

SFVcae_FV2014 10328 ATGCTAAAAAGGCTAAGGAACTTTTAGAAATGGACTATGATATTCATTGG 10377

||||||||||||||||.|||||||||.|||||||||||||||||||||||

SFVcae_LK3 10314 ATGCTAAAAAGGCTAAAGAACTTTTAAAAATGGACTATGATATTCATTGG 10363

SFVcae_FV2014 10378 GAACAATCTAAAGAAGATGCAAAATATTATGAAATAGAATATCATTGTAA 10427

|||||.||||||||||||.|..|.||||..||||||||||||||||||||

SFVcae_LK3 10364 GAACAGTCTAAAGAAGATTCTCAGTATTTCGAAATAGAATATCATTGTAA 10413

SFVcae_FV2014 10428 AATGTGTATGACTGTTATTCATGAGCCTATGCCTGTTTATTTTGATGAAA 10477

||||||||||||||||||||||||||||||||||||.|.||.||||.|||

SFVcae_LK3 10414 AATGTGTATGACTGTTATTCATGAGCCTATGCCTGTCTCTTATGATAAAA 10463

SFVcae_FV2014 10478 AAACTGGACTATGGATTAAAATGGGACCCCTTCGGGGTGATTTAGGATCA 10527

||||||||||.|||||||||||||||||.|||.|.||.||..||||.|||

SFVcae_LK3 10464 AAACTGGACTTTGGATTAAAATGGGACCTCTTAGAGGAGACATAGGCTCA 10513

SFVcae_FV2014 10528 GTAGTACATACTTGCAGGAGACATTATGAACGATGTATGTCTGCCCTTCC 10577

|||||.|||||.||.||.|||||||||||..|||||||||||||||||||

SFVcae_LK3 10514 GTAGTGCATACATGTAGAAGACATTATGAGAGATGTATGTCTGCCCTTCC 10563

SFVcae_FV2014 10578 TAGTTCAGGAGAACCTCTCAAACCCAGAGTGCGGGCTAATCCTGTCAGAA 10627

|||.||.|||||||||||||||||||||||.||||||||||||||.||||

SFVcae_LK3 10564 TAGCTCGGGAGAACCTCTCAAACCCAGAGTCCGGGCTAATCCTGTTAGAA 10613

SFVcae_FV2014 10628 GATATAGAGAGAAGTCACTCATCGTTGCGGATCGGCCTAAACGCTCCCGA 10677

||||.||||||||||||||||||||||||||||||||.|||||||||.||

SFVcae_LK3 10614 GATACAGAGAGAAGTCACTCATCGTTGCGGATCGGCCAAAACGCTCCAGA 10663

SFVcae_FV2014 10678 TGGGGTGTGGCCCCTCGCGAACAGCCCAATACTTCCAGTAGTGACGCCAT 10727

|||||||||||||||||.|||||||||||||||||||||.||||||||||

SFVcae_LK3 10664 TGGGGTGTGGCCCCTCGGGAACAGCCCAATACTTCCAGTGGTGACGCCAT 10713

SFVcae_FV2014 10728 GGCCCTTATGCCAGGACCATGCGGGCCCTACAATATGGACCCTCCTGGAT 10777

||||||||||||||||||||||||.||||.||||||||||||||||||||

SFVcae_LK3 10714 GGCCCTTATGCCAGGACCATGCGGCCCCTTCAATATGGACCCTCCTGGAT 10763

SFVcae_FV2014 10778 GCTTACTGGAGAGGGTACCGGGATCAGAACCTGGAACCTCCGAAATGGCT 10827

|||||||||||||||||||.||||||||||||||||||||||||||||||

SFVcae_LK3 10764 GCTTACTGGAGAGGGTACCAGGATCAGAACCTGGAACCTCCGAAATGGCT 10813

SFVcae_FV2014 10828 GTGGCTATGTCAGGAGGATCCTTCTGGGAGCAAGTATACCGGGACTCAAT 10877

.||||||||||.||||||.||||||||||.||||||||||||||||||||

SFVcae_LK3 10814 TTGGCTATGTCTGGAGGACCCTTCTGGGAACAAGTATACCGGGACTCAAT 10863

***Tas* |**

SFVcae_FV2014 10878 TTCTGGTCCCCCCACTGGGTCAAGT**TAA**ACTTAGGCTATATCAAAACTTA 10927

|||||||||||||||||||.|.|||.|||.|||||||||||||.||..||

SFVcae_LK3 10864 TTCTGGTCCCCCCACTGGGCCTAGTGAAAAT**TAG**GCTATATCAGAATCTA 10913

SFVcae_FV2014 10928 ACTGTTGTATATATTTGTCAATCTGTAGATCCATGGGAAAATGAGAATCC 10977

||||||||||||||||||||||||.|||||||||||||.|||||||||||

SFVcae_LK3 10914 ACTGTTGTATATATTTGTCAATCTATAGATCCATGGGAGAATGAGAATCC 10963

SFVcae_FV2014 10978 AACAGGTGGTCGAAGAGACCCCACCAGAAGGTATGGCTGTAGAATTGCTT 11027

||||||||||||||||||||||||.|||||||||||||||||||||||.|

SFVcae_LK3 10964 AACAGGTGGTCGAAGAGACCCCACTAGAAGGTATGGCTGTAGAATTGCAT 11013

SFVcae_FV2014 11028 GTGATCCTGTATATTGTGTAAAAATTGTTTGGGAAGGAAACTTCTGGGAT 11077

|||||||||||||||||||.||||||||||||||||||||..||||||||

SFVcae_LK3 11014 GTGATCCTGTATATTGTGTGAAAATTGTTTGGGAAGGAAATCTCTGGGAT 11063

SFVcae_FV2014 11078 AAAAAGGACCAACCTTGTTGGTTAATTAGACTTAAAGAAGGACATAATCA 11127

||||||||.||||||||||||||.|||||.||||||||||||||||||||

SFVcae_LK3 11064 AAAAAGGATCAACCTTGTTGGTTGATTAGGCTTAAAGAAGGACATAATCA 11113

SFVcae_FV2014 11128 TGGTGCAGAAGAATTGTCTCAAGAGGATATTAAAATCTTAAGTGAGTCCA 11177

|||||||.|||||.|.||||||...|||||||||||.|||.|.||.||.|

SFVcae_LK3 11114 TGGTGCAAAAGAACTATCTCAAAGAGATATTAAAATTTTAGGGGAATCTA 11163

SFVcae_FV2014 11178 GACCATACCCATATGGATCTATTGGACAATGTGCAAAATTACAATATGCT 11227

||||||||||||||||....||||||||||||.|||||||.||||||||.

SFVcae_LK3 11164 GACCATACCCATATGGCCTCATTGGACAATGTCCAAAATTGCAATATGCA 11213

SFVcae_FV2014 11228 GTACAGGTTAAGATGAGAGTTGACAAAGCACCTCTTACAGCCAAAGTTTT 11277

.||||.||.||.|||||||||||.||.||.||.|||||.||.||||||||

SFVcae_LK3 11214 ATACAAGTAAAAATGAGAGTTGATAAGGCTCCACTTACTGCTAAAGTTTT 11263

SFVcae_FV2014 11278 GTCAATAAAGGCATTACATTTCCATCGTTGGAACATATGCCAACCAGAAA 11327

..||.||||.|||||||||||||||||.|||||.||||||||...|||||

SFVcae_LK3 11264 AGCAGTAAAAGCATTACATTTCCATCGCTGGAATATATGCCAGAGAGAAA 11313

SFVcae_FV2014 11328 ACCCTGGAATTGGAGAAGGATATTCTCCTTCTGGATATACTCAAGCTCTA 11377

|.||.|||||||||||||||||||.|||.|||||||||||||||||.|||

SFVcae_LK3 11314 ATCCCGGAATTGGAGAAGGATATTTTCCCTCTGGATATACTCAAGCACTA 11363

**| LTR**

SFVcae_FV2014 11378 AAAGCTTATGGACCTCAGCATGGAAGTGTCGAGGAGAGGGTGTGGCTGAC 11427

||||||||||||||||||||||||||||.||||.|||||||||||||||.

SFVcae_LK3 11364 AAAGCTTATGGACCTCAGCATGGAAGTGCCGAGCAGAGGGTGTGGCTGAT 11413

SFVcae_FV2014 11428 ATCCACTAGAATGACAGGCCCCCAGGAAGATGATTATTGGAAAGATGCCT 11477

||||||||.|||...|||||||||||||.|.||||||||||.|||.||.|

SFVcae_LK3 11414 ATCCACTAAAATAGTAGGCCCCCAGGAAAAGGATTATTGGAGAGACGCAT 11463

SFVcae_FV2014 11478 ATCGATGGGGATATTTCCCTTTGGTCCCAAATAAACATCATCCTGGGTGG 11527

|.||.||||||||||||||.||.||.||.|||||.|||||||||||||||

SFVcae_LK3 11464 ACCGTTGGGGATATTTCCCCTTAGTTCCCAATAAGCATCATCCTGGGTGG 11513

SFVcae_FV2014 11528 ACTAGATATTTAACTAAATTTAAGATCTCTAGATTCTCCACTCCTGCTGA 11577

||||||.||||||||||.||.|||||.|||||||||.|||||||||||||

SFVcae_LK3 11514 ACTAGACATTTAACTAAGTTCAAGATTTCTAGATTCGCCACTCCTGCTGA 11563

SFVcae_FV2014 11578 TATCCAAAAGATTACAGATGAACTTCTCCCTAGAGGAGCAAGCATTGTTA 11627

.||||||||||||...||||||||||||||||||||||||||||||||||

SFVcae_LK3 11564 CATCCAAAAGATTGTTGATGAACTTCTCCCTAGAGGAGCAAGCATTGTTA 11613

SFVcae_FV2014 11628 CAGCTGATGGATCAAAGTATGAAAGCACTAGAAAAGTGCATTTGGTTAAT 11677

|||||||||||...|.|||||||||||||||.||.||.|||||.||||||

SFVcae_LK3 11614 CAGCTGATGGAAATAGGTATGAAAGCACTAGGAAGGTACATTTAGTTAAT 11663

SFVcae_FV2014 11678 GAAGGAACTCTCAAAGAATACCAAGACAAAAATAGAGAAATAGAAGAAAA 11727

||||||||.||..||||.||||||| |.||||||..|||||||||||||.

SFVcae_LK3 11664 GAAGGAACCCTTGAAGAGTACCAAG-CTAAAATAAGGAAA**TAG**AAGAAAG 11712

SFVcae_FV2014 11728 ATATGGATGTGGATGTTGTCTGTCCTCAGATAGTGATGATGATGATTATT 11777

|||||||||||||||||||||.||.|||||||||||||||||.|||||||

SFVcae_LK3 11713 ATATGGATGTGGATGTTGTCTTTCTTCAGATAGTGATGATGAGGATTATT 11762

***bet* |**

SFVcae_FV2014 11778 CCGAGGATACACCTGATACAGAAAGTACTAGTGTTGAAGAAGAC**TAA**AAT 11827

|.||.|||||.|||||.||.||||.|||.|||||.||.|||||.|||||.

SFVcae_LK3 11763 CTGAAGATACCCCTGACACTGAAACTACAAGTGTAGAGGAAGATTAAAAC 11812

SFVcae_FV2014 11828 GCAGGGTACAGTGTTGTTCATTTACATAATCTGCTTAGCAACTGCTTATG 11877

.||||||||||||||.||.|||||||||||||||||||||||||||||||

SFVcae_LK3 11813 ACAGGGTACAGTGTTATTTATTTACATAATCTGCTTAGCAACTGCTTATG 11862

SFVcae_FV2014 11878 CAATAAGAGTGATTCAGTATATTGTTTAGGAATAAGATATAGTTTAATAG 11927

|.. ||||.|||||||||||||||||||||||||||||||||||||..||

SFVcae_LK3 11863 CTC-AAGAATGATTCAGTATATTGTTTAGGAATAAGATATAGTTTATAAG 11911

SFVcae_FV2014 11928 TAGTTAATCCTTAGGGAGTATTTGGTGGAAACGACTAAGTGACAC-AAGT 11976

.|||||||||||||||||||||||||||||||||||.|||||||. ||||

SFVcae_LK3 11912 AAGTTAATCCTTAGGGAGTATTTGGTGGAAACGACTGAGTGACATGAAGT 11961

SFVcae_FV2014 11977 TTATTCACCATACTCTCAATAGGAGCCACTAGTTGAGCCTGTGTGTTCAA 12026

||||||||||||||||||||||||||||||||||||||||||||||||||

SFVcae_LK3 11962 TTATTCACCATACTCTCAATAGGAGCCACTAGTTGAGCCTGTGTGTTCAA 12011

SFVcae_FV2014 12027 ATCCATGCTCAGCTAAAGTGACTCCCTTTTAGTTTCACTTTTAGGTTAAG 12076

||||||||||||||||||||||||||||||||||||||||| |||.||||

SFVcae_LK3 12012 ATCCATGCTCAGCTAAAGTGACTCCCTTTTAGTTTCACTTT-AGGATAAG 12060

SFVcae_FV2014 12077 -ATAGATATAGATTCCATATAATCCTAAGGGAGTATGTGGACCTTCTTGT 12125

|||| ||||..|..|||||||||||||||||||||||||||||||||||

SFVcae_LK3 12061 TATAG-TATAAGTATCATATAATCCTAAGGGAGTATGTGGACCTTCTTGT 12109

SFVcae_FV2014 12126 TAGGAATTAGTTTAAGATAGTCCACAGCTCCCTTCTTTTTAAGTTCAAGA 12175

||||||||||||||||||||||||||||||||||||||||.|||||.||.

SFVcae_LK3 12110 TAGGAATTAGTTTAAGATAGTCCACAGCTCCCTTCTTTTTGAGTTCTAGT 12159

SFVcae_FV2014 12176 CTATGTATAGTTTGTTGGCTCATAACAGATAAAGTGCTCATAAGACAGGA 12225

||.|||..|||||||||||||||| ||||||||||||||||.|.||||||

SFVcae_LK3 12160 CTTTGTTAAGTTTGTTGGCTCATA-CAGATAAAGTGCTCATTAAACAGGA 12208

SFVcae_FV2014 12226 AACCGCAACCGGGTAAAGGTTAGCACAGTTTGTTAAGCTAGTCGTTACCC 12275

|||||||||||||||||||||||||||||...|||||||||..|||||.|

SFVcae_LK3 12209 AACCGCAACCGGGTAAAGGTTAGCACAGTAAATTAAGCTAGCAGTTACTC 12258

SFVcae_FV2014 12276 AAGAGCCCGGTAAGCATTTAAGTGGTTCGAGTCTCTTTAATGCTGACGGA 12325

||||||||||||||||||.||||.||||||.||.||||||||||||||||

SFVcae_LK3 12259 AAGAGCCCGGTAAGCATTCAAGTAGTTCGAATCCCTTTAATGCTGACGGA 12308

SFVcae_FV2014 12326 TTGCTCTTTAGTGAAGTGATGTAATATGTTTTTGTGAATCAAAATGTGTT 12375

||||||||||||||.||||||||||.|||||||||.|....||||||||.

SFVcae_LK3 12309 TTGCTCTTTAGTGAGGTGATGTAATTTGTTTTTGTAATCTGAAATGTGTA 12358

SFVcae_FV2014 12376 TCTGAACAGGAAGTAAAACAAGAAAGGGAATGGCTAAACTTGTTTAAGCT 12425

|.||.|.|||||||...||||.||||||||||||||||||||||..||||

SFVcae_LK3 12359 TTTGTATAGGAAGTTGTACAAAAAAGGGAATGGCTAAACTTGTTACAGCT 12408

SFVcae_FV2014 12426 CAAGCAAACATTTAGCTCTTTCCTTTGCTTTTGGAGTTCGAGTCTTGTAA 12475

|.||||||||||||||..|||||||||||||||.|||||||.||||||||

SFVcae_LK3 12409 CGAGCAAACATTTAGCAATTTCCTTTGCTTTTGAAGTTCGAATCTTGTAA 12458

SFVcae_FV2014 12476 TTGCATTTTGAGCACTGTATTTAGAATAACTTAAGTATGGAAAAATCTCC 12525

|...|||||.|||||...|..||.|| |.||||||||||||||||||..|

SFVcae_LK3 12459 TAATATTTTAAGCACATGAACTATAA-AGCTTAAGTATGGAAAAATCCTC 12507

SFVcae_FV2014 12526 AAGTATGAGTCACGAGATGCTTGGCTCACTGCGTTGGACGACTGGATAGA 12575

|||||||||||||||||||.||||||||||||||||||||||||||.|||

SFVcae_LK3 12508 AAGTATGAGTCACGAGATGTTTGGCTCACTGCGTTGGACGACTGGAAAGA 12557

SFVcae_FV2014 12576 AGCTTCAACAGTCGGGACAGCATCTCGAAGAAGGCCTCCAGAGTGAAAGA 12625

|||||||||||||||||||||||||||||||||||||||.||.|||||||

SFVcae_LK3 12558 AGCTTCAACAGTCGGGACAGCATCTCGAAGAAGGCCTCCGGAATGAAAGA 12607

SFVcae_FV2014 12626 GTGGAAAT-GAAATCTCCTCATTCAGAGTGCCTTCTTTTT-AACTTTAGG 12673

||..|||| |||.|||||||||||||||.||||||||||. ||.||||||

SFVcae_LK3 12608 GTAAAAATTGAAGTCTCCTCATTCAGAGAGCCTTCTTTTGGAATTTTAGG 12657

SFVcae_FV2014 12674 TAGAATATAGTTTCCAGTAGGATAAACTTTTGTACTAGCTGATAGATAGG 12723

||||..|.||||||.|.|||.|||||||||||||..|||.||||||||||

SFVcae_LK3 12658 TAGAGAAAAGTTTCTAATAGAATAAACTTTTGTATCAGCAGATAGATAGG 12707

**U3 | R**

SFVcae_FV2014 12724 ATATATAATCTCTGCTTTAGATTGTACGAGAGCTCTTCACTACTCGCTGC 12773

||||||||||.|||||||||||||||||.||||||..||||.||||||||

SFVcae_LK3 12708 ATATATAATCCCTGCTTTAGATTGTACGGGAGCTCACCACTGCTCGCTGC 12757

SFVcae_FV2014 12774 GTCGAGAGTGTTTGAGTCTCTCCAGGCTTGGTAAGATATAAACTTTGGTA 12823

||||||||||||.|||||||||||||||||||||||||.|| ||||||||

SFVcae_LK3 12758 GTCGAGAGTGTTCGAGTCTCTCCAGGCTTGGTAAGATAGAA-CTTTGGTA 12806

SFVcae_FV2014 12824 TTCTCTGTATTATCTATGATCCAATAATACTCTGCTTATAGATTGTAATG 12873

||||.||||||.| |||||||||||.||||||||||||||||||||||||

SFVcae_LK3 12807 TTCTATGTATTTT-TATGATCCAATTATACTCTGCTTATAGATTGTAATG 12855

SFVcae_FV2014 12874 GGCAATGGCAATGCTTAAT-AATTAATGAAGTTTATGGTGAATTAAGTTC 12922

||||||||||||||||.|| |||.|||||. |||||||||||||||||||

SFVcae_LK3 12856 GGCAATGGCAATGCTTTATCAATGAATGAT-TTTATGGTGAATTAAGTTC 12904

**R | U5**

SFVcae_FV2014 12923 ATATATGTTTAAAGAAGTTTATCAATAAACCGACTTAATTCGAGAACCAG 12972

|||||||||||||||||||||.||||||||||||||||||||||||||||

SFVcae_LK3 12905 ATATATGTTTAAAGAAGTTTAACAATAAACCGACTTAATTCGAGAACCAG 12954

SFVcae_FV2014 12973 ATTTATTAGTATTGTCTCTTTCTATACTTAA-GTAAAGTGAAAGGAATTG 13021

|||||||||||||||||||||||||||||.| |.||||||||||||.|||

SFVcae_LK3 12955 ATTTATTAGTATTGTCTCTTTCTATACTTTATGCAAAGTGAAAGGAGTTG 13004

SFVcae_FV2014 13022 TGTATTAGCCTTGCTTAAGAAAATCATTTAACAGTATAAGTGTGTACTAC 13071

|.|||||||||||||||..|.|..|||.||...||||||||||..|.|||

SFVcae_LK3 13005 TATATTAGCCTTGCTTATAAGAGCCATCTAGTGGTATAAGTGTAGATTAC 13054

SFVcae_FV2014 13072 ACTTATCATAAGGGGTGATATT-TCTAAGGATAATCAATACACAATAATC 13120

||||||||||||.||||..||| |.|||||||||||||||.||||.|.||

SFVcae_LK3 13055 ACTTATCATAAGAGGTGGAATTCTTTAAGGATAATCAATATACAAAATTC 13104

SFVcae_FV2014 13121 CATGACA 13127

||.||||

SFVcae_LK3 13105 CACGACA 13111
